# Systems Biology as an Integrated Platform for Bioinformatics, Systems Synthetic Biology, and Systems Metabolic Engineering

**DOI:** 10.3390/cells2040635

**Published:** 2013-10-11

**Authors:** Bor-Sen Chen, Chia-Chou Wu

**Affiliations:** Laborotary of Control and Systems Biology, Department of Electrical Engineering, National Tsing Hua University, HsinChu 30013, Taiwan

**Keywords:** systems biology, bioinformatics, systems synthetic biology, systems metabolic engineering

## Abstract

Systems biology aims at achieving a system-level understanding of living organisms and applying this knowledge to various fields such as synthetic biology, metabolic engineering, and medicine. System-level understanding of living organisms can be derived from insight into: (i) system structure and the mechanism of biological networks such as gene regulation, protein interactions, signaling, and metabolic pathways; (ii) system dynamics of biological networks, which provides an understanding of stability, robustness, and transduction ability through system identification, and through system analysis methods; (iii) system control methods at different levels of biological networks, which provide an understanding of systematic mechanisms to robustly control system states, minimize malfunctions, and provide potential therapeutic targets in disease treatment; (iv) systematic design methods for the modification and construction of biological networks with desired behaviors, which provide system design principles and system simulations for synthetic biology designs and systems metabolic engineering. This review describes current developments in systems biology, systems synthetic biology, and systems metabolic engineering for engineering and biology researchers. We also discuss challenges and future prospects for systems biology and the concept of systems biology as an integrated platform for bioinformatics, systems synthetic biology, and systems metabolic engineering.

## 1. Introduction

The Human Genome Project and high-throughput experimental methodologies such as microarray chromatin-immunoprecipitation DNA chips (ChIP-chip) have led to the development of biology as an increasingly information-rich science encompassing transcriptomes, proteomes, metabolomes, interactomes, and so forth [1,2]. Some have suggested that systems biology is nothing more than a new name for integrative physiology, which has been practiced for the past 50 years or more. Because of these novel technologies, biologists have been able to collect data at a rate that was unimaginable a decade ago. The context of biology has profoundly changed over the past 20 years. These changes provide a powerful new framework for systems biology that moves it far beyond classical integrative physiology. A systems biology approach implies that every system of any level of biological systems can be analyzed with respect to the system’s structure, in particular, in terms of its dynamics, method of control, and method of system design. Systems biology involves genomic, transcriptomic, proteomic, and metabolic investigations from a systematic perspective. As a result, systems biology has become the frontier of modern biological research; large amounts of new omics data cannot be understood without a network or systems viewpoint and without highly sophisticated computational analyses [3,4,5,6,7,8,9,10,11]. 

**Figure 1:**
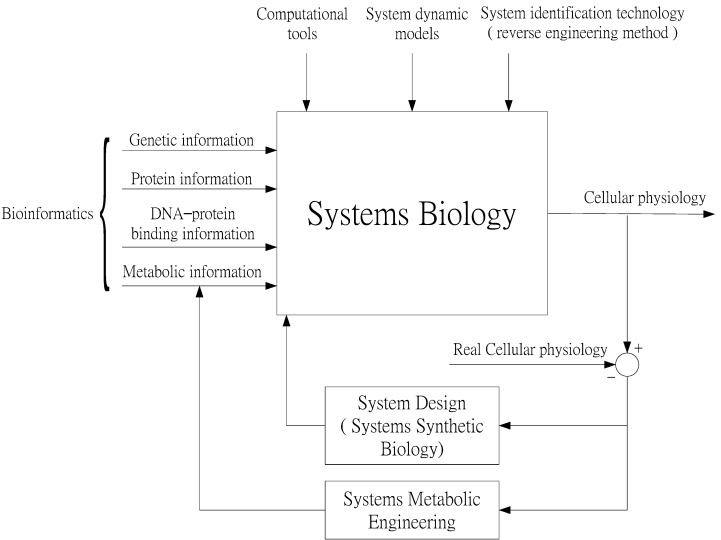
The role of systems biology as an integrated platform in modern biological research Systems biology integrates information on genetics, proteins, DNA-protein binding, and metabolism with system dynamics modeling and system identification technology to develop models and mechanisms for the interpretation of phenotypes or behaviors of cellular physiology. Since large-scale data sets need to be mined, powerful computational tools are necessary. Based on system models and mechanisms in systems biology, synthetic genetic circuits are designed to investigate specific desired cellular behaviors of cellular physiology. Discrepancies between real and desired cellular behaviors are used as feedback to adjust system models and mechanisms. Systems biology is thus positioned to play the role of integrated platform for bioinformatics, systems synthetic biology, and systems metabolic engineering.

The role of systems biology in modern biological research (Figure 1) requires powerful computational tools to mine large-scale data sets of information on genetics, proteins, DNA–protein binding, metabolism, and so forth. These tools are used to construct dynamic system models for the interpretation of specific mechanisms of some cellular phenotypse (or behaviors) from a system (or network) perspective [12,13,14,15,16,17]. To construct a dynamic system model of biological networks from omics data, system identification technologies (*i.e.*, reverse-engineering schemes) are needed to estimate the parameter values of dynamic models and the order of biological networks [18,19,20,21,22,23,24,25,26,27,28,29,30,31,32,33,34,35,36,37,38,39,40,41,42,43,44]. Synthetic biology metabolic engineering has recently been developed for designing synthetic genetic networks for the production of specific cellular functions in host cells [45,46,47,48,49,50,51]. Based on system models and mechanisms in systems biology, synthetic genetic circuits and metabolic engineering pathways can be designed to investigate cellular behaviors [52,53,54,55,56,57,58,59,60,61,62,63]. These synthetic biological technologies can be employed to investigate the models and mechanisms of systems biology. Discrepancies between the real behavior of synthetic genetic networks and the desired behavior predicted by the models and mechanisms of systems biology can be fed back to modify the models through methodologies of systems synthetic biology and systems metabolic engineering. Based on the role of systems biology (Figure 1), this review describes current developments in bioinformatics, systems synthetic biology, and systems metabolic engineering. It discusses how systems biology can serve as an integrated platform for bioinformatics, systems synthetic biology, and systems metabolic engineering in the future.

Bioinformatics is crucial in genome-wide analyses for understanding cell physiology at different cellular levels (*i.e.*, genome, transcriptome, proteome, and metabolome levels) [12,13]. The various disciplines of bioinformatics provide invaluable information on the global cellular status for systems biology, systems synthetic biology, and systems metabolic engineering, as well as a thorough analysis of the cell. Genomic information in bioinformatics represents the whole genetic makeup of the organism [12,13,14,15,16,17], and comparative genomic analysis may contribute to systems synthetic biology or systems metabolic biology for targeting and engineering genetic circuits to create desirable cellular phenotypes. Transcriptome profiling uses DNA microarrays to decipher the expression levels of thousands of genes under various biological conditions [14,15]. The results can be used to select candidate genes for modification based on systematic analysis of regulatory genes in response to genetic variations and environmental changes, or to identify novel factors for the enhancement of heterologous product secretion in metabolic pathways [64,65,66,67,68]. Proteome profiling is also useful in obtaining transcriptome-profiling data at the protein level. The metabolome comprises the entirety of information on metabolites present within and/or outside the cell under specified conditions [61,62,63]. It is expected to contribute significantly to the understanding of the cell and of synthetic circuit engineering in its metabolic pathways. In this paper, recent advances in the application of bioinformatics to systems synthetic biology and systems metabolic engineering through systems biology are reviewed using specific examples.

Although bioinformatic information (e.g., data on genetics, protein binding, and metabolism) is available, several stages of systems biology are required to help us understand via system dynamics modeling the underlying molecular mechanisms of genetic regulatory (GR) networks [18,19,20,21,22,23,24], protein–protein interaction (PPI) networks [25,26,27,28,29], and metabolic networks [61,62,63] under various biological conditions. At the first stage, a putative GR or PPI network is created by large-scale integration of knowledge such as information from publications and databases, and high-throughput data (from data mining or deep curation). Based on this network and dynamic modeling, the actual GR or PPI network of cellular physiology can be identified with system identification methods (reverse-engineering scheme [34]) by using specific microarray gene or protein expression data [18,19,20,21,22,23,24,25,26,27,28]. For example, GR networks (GRNs) have been constructed by dynamic modeling via microarray data for cell cycles [18,23,24], environmental stressors [28,44], photosynthesis [69], aging [34], and cancer [39]. PPI networks have been constructed for cancer [3,9,33,39,70], inflammation [41], biofilm formation [43], and infection by *Candida albicans*. Comparison of PPI networks between healthy and cancer cells can provide network markers for the investigation of the systematic mechanism of cancer [70]. The integration of cellular networks of GRs and PPIs provides deeper insight into actual biological networks and is more predictive than an approach without integration [71]. A systematic and efficient method to integrate different kinds of omics data for the construction of integrated cellular networks via microarray data have been provided based on coupling dynamic models and statistical assessments [44]. This method has been shown to be powerful and flexible, and can be used to construct integrated networks at different cellular levels to investigate cellular machinery under different biological conditions and for different species. Coupling dynamic models of the whole integrated cellular network is very useful for theoretical analyses and for further experiments in the field of network biology and synthetic biology. 

In short, synthetic biology is the engineering of biological systems to fulfill a particular purpose. It is possible to build living machines from off-the-shelf genetic devices by employing many of the same strategies that electrical engineers use to manufacture computer chips [47,48,49]. The main goal of this nascent field is the design and construction of biological systems with desired behaviors [51]. Synthetic biology envisions the redesign of natural biological systems as well as the construction of functional “genetic circuits” by using a set of powerful biotechniques for the automated synthesis of DNA molecules and their assembly into genes and microbial genomes [47]. Synthetic biology is predicted to have important applications in biotechnology, metabolic engineering, and medicine. It may revolutionize how we conceptualize and approach the engineering of biological systems [49]. As illustrated in Figure 1, synthetic genetic circuits can furthermore be employed to confirm network mechanisms derived using systems biology methods, and can be used as feedback for their improvement or revision. Synthetic biology is therefore expected to contribute significantly to a better understanding of the functioning of complex biological systems such as metabolic pathways. 

However, the development of synthetic gene networks is still difficult. Most newly created gene networks are nonfunctional because of intrinsic parameter fluctuations, environmental disturbances, and functional variations in the intra- and extracellular context. For this reason, the design method based on dynamic models for robust synthetic gene networks has become an important topic in synthetic biology [52,53,54,55,56,57,58,59,60,68]. These system-dynamics-based design methods for synthetic biology lead to systems synthetic biology.

Heterologous genes have previously been combined into pathways in metabolic engineering, generating a myriad of non-native biochemical products, including isoprenoids, hydroxyacids, biofuels, polypeptides, and biopolymers [64,65,66,67]. Synthetic biologists developed synthetic tools to engineer genetic devices capable of performing complex biological functions such as sensing cell states, counting cellular events, and implementing computational logic. These tools have been applied to the modification and control of metabolic pathways in several organisms. They consist of one or more parts that have been combined to perform a complex function, and provide metabolic engineers with novel ways of exerting cellular control over heterologous production pathways. Some synthetic biological devices with potential relevance in metabolic engineering include orthogonal inducible promoters, light-sensitive promoters, state sensors, spatiotemporal controllers, and logic gates, as well as promoter and ribosome binding site (RBS) libraries [36,37]. Since metabolic engineering seeks to control cellular metabolism and manipulate through heterologous pathways to maximize production of a desired molecule, metabolic engineers are need elegant methods for gathering bio-information about cells, their environment, and modulating gene expression in responses [37,61,62,63]. Hence, devices of synthetic biology promise to be a useful addition to the metabolic engineering toolbox. Some synthetic devices have already been used to increase product titers. However, many remain largely untested in an industrial setting, and the complexity of biology makes their application a feat of engineering [36,37,72]. From the systems biology perspective, continuous work with these devices can help elucidate design rules or aid the development of system dynamics models that facilitate their integration into metabolic industrial processes [36,37,72], and thereby lead to the development of systems metabolic engineering.

Several mathematical techniques based on systems biology have been developed to analyze the systematic properties of complex biological networks. For example, system sensitivity of a biological network in response to various parameter variations has been analyzed to determine the systematic properties that affect the robustness and fragility of a biological network. System sensitivity analysis not only can reveal the robust stability of a biological network against various perturbations, but may also provide information about the controllability of a biological network [7,8,9]. The system response ability of a biological network is a measure of response to environmental signals or disturbances [28,34]. From the system theory perspective, robustness to intrinsic system variation and the ability to respond to external stimuli are two important and complementary system characteristics in the evaluation of system performance [33,34]. A more biological system that is robust toward a large amount of intrinsic parameter fluctuations is less responsive to environmental disturbances, and vice versa. A systems biology investigation of the aging-related gene network via microarray data found that network robustness increases and network response ability decreases during the aging process [34]. The sensitivity of a biological genetic system to environmental molecular noise is considered as an indication of the noise-filtering ability of the gene network [42]. Systems biology allows the measurement of its system characteristics, as well as the capabilities of the signal transduction pathway [8] Similarly, flux amplification of the metabolic pathway can be estimated, by using both system dynamics models.

As nonlinear biological networks operate under different conditions of cellular homeostasis and homeodynamics, systems biology studies on complex biological models in the landscape of phenotypes are highly informative. These studies help discover possible equilibrium points (phenotypes) and dynamic behaviors, such as bifurcation, oscillation, robust stability, and phase drift to other equilibrium points (phenotype transition). Bifurcation analysis and phase-plane analysis of nonlinear dynamic networks can be useful in predicting system behavior of biological networks under intrinsic parameter changes. Through systems biology approach and dynamic modeling [38,39,40,41,42], network robustness and noise filtering ability can be improved via feedback, redundancy, and modular schemes. This is why there are so many feedback loops, redundant genes, and modular structures at different scales of biological networks. A unifying mathematical framework based on nonlinear stochastic dynamic models [73,74,75] was recently proposed to describe different levels of stochastic biological networks under different parameter fluctuations, genetic variations, and environmental disturbances [29,30,59]. The phenotype robustness criteria of biological networks in systems, evolutionary, ecological, and synthetic biology were investigated from a systematic perspective on the basis of robust stabilization and filtering ability. Network robustness of biological networks can confer intrinsic robustness toward intrinsic parameter fluctuations, genetic robustness for buffering genetic variations, and environmental robustness for resisting environmental disturbances. It was found that if the sum of intrinsic robustness, genetic robustness, and environmental robustness is less than or equal to the network robustness, then the phenotype is robust in different levels of biological networks in systems, evolutionary, ecological, synthetic, and metabolic biology. These phenotype criteria at different levels of biological networks are useful for the design of synthetic and metabolic systems. A systems biology approach based on dynamic models can clearly provide not only a systematic insight into behaviors at different levels of biological networks, but also a design platform to improve system robustness, filtering ability, and transduction ability of synthetic and metabolic system networks, which are discussed in detail in the following sections.

## 2. Systems Biology Approach to GRNs and PPI Networks via Bioinformatics Methodology

### 2.1. Construction of GRNs via Microarray Data

The construction of a GRN using a systems biology approach can be divided into two steps. The first step is construction of a potential (or putative) GRN from two high-throughput data sets, namely, a ChIp network and a mutant network. The ChIp data are based on experimental data published by Harbison *et al.* [14], who also used a genomic tiling array to identify the genomic region bound by transcription factors (TFs). The mutant data are the gene expression data matrix published by Hughes *et al.* [76] with different gene deletion mutants. In general, the potential GRN is suitable for all possible biological conditions. Therefore, the GRN for a specific biological condition needs to be confirmed using microarray gene expression data of the specific biological condition; that is, the real GRN is derived by pruning the potential GRN with specific microarray data. Let *x_i_*(*t*) denote the gene expression of the *i-*th target gene in the potential GRN. The dynamic equation for the regulation of the *i-*th gene is then modeled as [21,22]

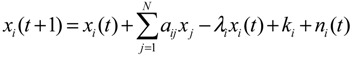
(2.1)
where *x_i_*(*t*) represents the mRNA expression level of target gene *i* at time *t*, *x_j_*(*t*) represents the regulation function of the *j*-th TF binding to the target gene *i*, *a_ij_* denotes the regulatory ability from the *j-*th regulatory gene to the *i-*th target gene (the positive sign indicates activation and the negative sign indicates repression), *λ_i_* indicates the degradation effect of the present time point on the next time point *t*+1, *k_i_* represents the basal level, and *n_i_*(*t*) is the stochastic noise due to model uncertainty and fluctuation of microarray data of the target gene. Expression of the *i-*th gene in (2.1) can be represented by the following regression equation [77]

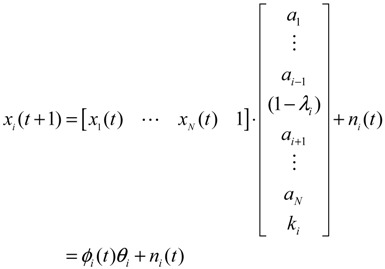
(2.2)
where *ϕ_i_* denotes the regression vector, which can be obtained from microarray data. *θ_i_* is the regulatory parameter vector of target gene *i*, which is to be estimated.

The regulatory parameter can be estimated from microarray data of the corresponding gene through the least-squares or maximum-likelihood parameter estimation [77] (known as the reverse-engineering method). After regulator parameters in *θ_i_* are estimated, the system order (*i.e.*, the number of regulatory genes) is determined by model comparison (e.g., Akaike’s information criterion (AIC)), and the *P*-value statistical method is employed to determine the significant regulatory genes for target gene *i.* This is done by pruning false-positive regulations in the potential GRN. That is, some *a_ij_* is pruned because of false positive deletion. Based on the dynamic model in (2.1), the true GRN can then be constructed one target gene at a time through microarray data. Using similar methods, GRNs for yeast cell cycles [18,23,24], cancer cell cycles [78], stress response [44], and inflammation [41] can be constructed.

### 2.2. Construction of PPI Networks

The construction of PPI network via a systems biology approach is also a two-step process. The first step is constructing a potential PPI network via data mining from literature and databases such as BioGRID, SGD, and GO [16,17]. As this is only a candidate network based on many biological conditions, the second step is pruning false positive PPIs by using a protein expression microarray of a specific biological condition. For a target protein *i* in the potential PPI network, the dynamic model of protein activity is [19,20]

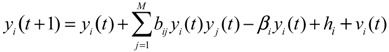
(2.3)
where *y_i_*(*t*) represents the protein activity level of the target protein *i* at time *t*, *b_ij_* denotes the interaction ability of the *j-*th interactive protein with the *i-*th protein, *y_j_*(*t*) represents the activity level of the *j-*th protein interacting with the target protein *i*, *β_i_* denotes the degradation effect of the protein, *h_i_* represents the basal activity level, and *ν_i_*(*t*) is the stochastic noise. The rate of PPI is proportional to the product of the concentrations of both proteins [7], that is, it is proportional to the probability of molecular collisions between them. The physical interpretation of Equation (2.3) is that the activity of target protein *i* at time *t*+1 is the combination of present protein activity, regulatory interactions with *M* interactive proteins, levels of basal protein from other sources and *M* interactive proteins in the cell, and stochastic noise, less the protein degradation of the present state. The PPI dynamic equation of target protein *i* in the potential PPI network can be represented by the following regression equation [77]:

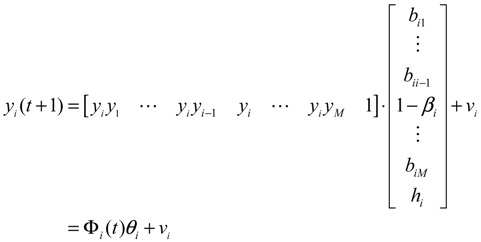
(2.4)


The interaction parameter *θ_i_* can be estimated from protein profile microarray data by least-squares or maximum-likelihood parameter estimation [77] (if protein profile microarray data are unavailable, ten mRNA microarray data could be used to replace them, with some modification [19,20]). By using AIC to prune false positive interactions, the real PPI network can then be constructed one target protein at a time by following the above two-step procedure. Some dynamic metabolic pathways [20] and PPI networks of cancer [39] and inflammation [41] have recently been constructed by using the microarray data and AIC method. Comparison of PPI networks between healthy and cancer cells can provide network-based biomarkers for molecular investigation and diagnosis of cancer [70].

### 2.3. Construction of Integrated GRN and PPI Cellular Networks

**Figure 2:**
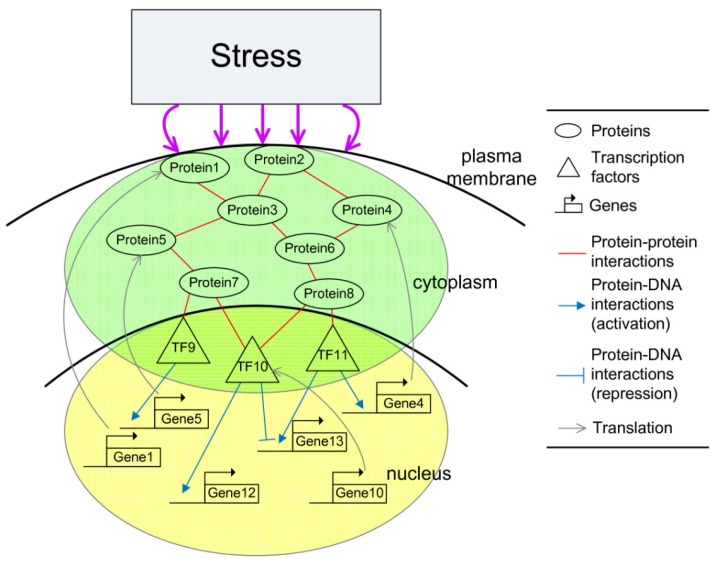
Schematic diagram of the integrated cellular network. The integrated cellular network consists of two subnetworks. The signaling regulatory pathway (green) contains protein–protein interaction (PPIs). The gene regulatory network (yellow) contains transcription regulations. The transcription factors serve as the interface between the two subnetworks.

Living organisms have evolved complex mechanisms to respond to changes in environmental conditions. This is the case even in unicellular organisms like the yeast *Saccharomyces cerevisiae*. Such environmental changes, commonly termed as “stress”, are harmful or even lethal to the survival of the cells, especially of microorganisms whose environment is very changeable. Cellular responses to stresses require complex cellular networks of sensing and signal transduction pathways, as well as metabolic pathways to adapt cell growth and proliferation for adjustments of gene expression programs, metabolic activities, and other features of the cell (see Figure 2). 

These regulatory systems and signal and metabolic pathways can therefore be suitably described by an integrated cellular network of transcription regulation and PPIs. The flowchart for constructing the integrated cellular network is shown in Figure S1. The same two steps already described apply (construction of a potential cellular network and pruning of false positives via specific microarrays of gene expression and protein expression data). Several kinds of omics data and database information were integrated, including data on microarray gene expression and protein expression, regulatory associations between TFs and genes, and PPI. From these data, the potential integrated GRN and PPI networks are retrieved. In the second step, based on the dynamic model of integrated gene/protein interactions in the potential cellular network, we find that [71]

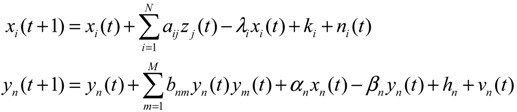
(2.5)
where the regulation function *z_j_*(*t*) is modeled as the sigmoid regulation function of *y_j_*(*t*), that is, for the protein activity profiles of transcription factor *j* [71],

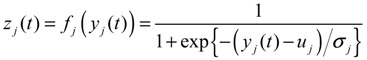
(2.6)
where *f_j_*(*y_j_*(*t*)) denotes the sigmoid function, *u_j_* and *σ_j_* represent the mean and deviation of protein activity level of TF *j*, and *α_n_* denotes the translation effect from mRNA *x_n_*(*t*) to protein *y_n_*(*t*).

Interaction and coupling among genes and proteins in the integrated cellular network in (2.5) are described as follows. If some TFs *y_j_*(*t*) at the end of the signal regulatory pathway regulate their target genes through the regulation function *z_j_*(*t*) = *f_j_*(*y_j_*(*t*)) in (2.6), then the regulatory genes influence their corresponding proteins in signal and metabolic pathways through translation effect *α_n_x_n_*(*t*). The interplay between genes and proteins can be seen from their coupling dynamic equations in (2.5) and **Figure 2**. Here, the TFs serve as the interface between the signaling regulatory pathway and gene regulatory network. In other words, the interplay between transcription regulation and PPIs constitutes the framework of the integrated cellular network.

**Figure 3:**
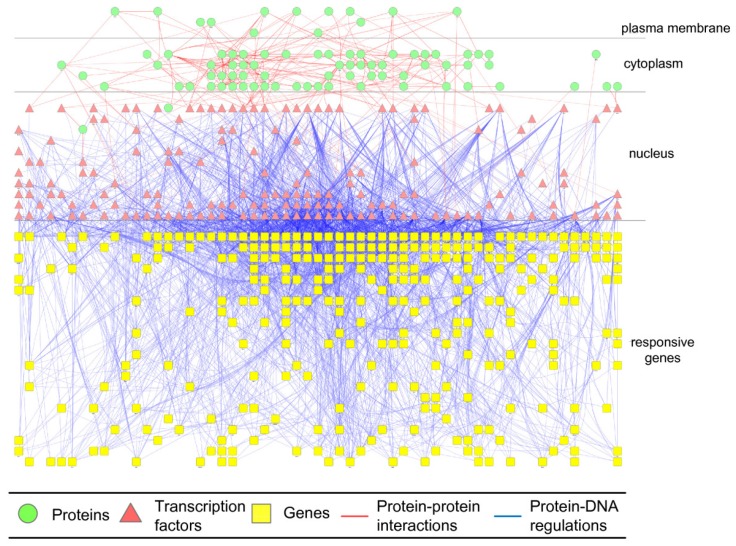
The *S. cerevisiae* integrated cellular network under hyperosmotic stress. By following the schematic diagram of an integrated genetic regulatory network (GRN) and PPI cellular network in Figure S1, the integrated cellular network of signaling regulatory pathway and GRN for hyperosmotic stress in *S. cerevisiae* is identified by dynamic modeling and data mining. Receptor proteins in the plasma membrane, signal regulatory pathways in the cytoplasm, and transcription factors and GRNs in the nucleus are used to construct an integrated cellular network for *S. cerevisiae* under hyperosmotic stress.

Based on the dynamic coupling equation in (2.5), the potential integrated cellular network can be linked through the regulatory parameter *a_ij_* between genes and their possible regulatory TFs and through the translation parameter *α_n_* for gene expression to protein expression. The potential signaling or metabolic pathways can be linked through the interaction parameter *b_nm_* between possible interaction proteins. Since omics data on the potential gene regulatory network and potential signaling or metabolic pathway only indicate possible TF-gene regulation and protein interactions, they should be confirmed using microarray data of gene and protein expressions. In particular, values of *a_ij_* and *b_nm_* in (2.5) should be identified and validated by least-squares estimation via microarray data in a specific biological condition or phenotype [71]. Significant regulations and interactions between genes and proteins were detected using model selection methods such as AIC and statistical assessments like such as Student’s t-test [77]. Based on the interface between gene regulatory and signal/metabolic networks (*i.e.*, transcription factors) in a specific biological condition, the two networks are coupled together to form the integrated cellular network. The integrated cellular network for *S. cerevisiae* under hyperosmotic stress is shown in Figure 3. After the construction of GRN and PPI network from microarray data and bioinformatic method, various system characteristics of the biological network are estimated or measured using systems biology methods in the following sections so that these systematic methodologies can be applied the systematic design of systems synthetic biology and systems metabolic engineering.

### 2.4. Network Robustness and Sensitivity Estimation via Microarray Data Using a Systems Biology Approach

After the GRNs or PPI networks or their integrated cellular networks are constructed by dynamic modeling using microarray data, some characteristics of these biological networks need to be estimated to gain insight into their systematic mechanisms. “Robustness”, the network ability to maintain systematic performance under intrinsic perturbations, and “response ability”, the network sensitivity to respond to external stimuli or to transduce them to downstream regulators, are two important complementary or antagonistic system characteristics that must be considered when discussing biological network performance. However, these systematic features cannot be measured directly for all network components in the experimental procedure. Even biological processes such as development, differentiation, tumorigenesis, and aging are increasingly being described in terms of temporal ordering of highly orchestrated transcriptional programs [33,34]. The term robustness is encountered widely in diverse scientific fields, from engineering and control theory [73,74,75] to dynamic systems [79] and biology [80]. It is important to note that robustness describes a relative property, not an absolute one, as no system can maintain stability in all functions when it is perturbed. In other words, robustness is not immutable. 

Let the GRN of interest consist of *N* genes. After parameter identification via microarray data, the GRN in (2.1) can be represented by the following linear discrete-time dynamic system [33,34]

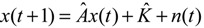
(2.7)

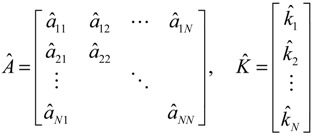

where the state vector *x*(*t*) = [*x*_1_(*t*) ⋯ *x_N_*(*t*)]*^T^* stands for the discrete-time mRNA expression levels of total *N* genes at times *t* = 1, 2, ..., *K*. The system matrix *Â* denotes gene interactions in the gene network estimated by microarray data, that is, *â_ij_* denotes the estimated interaction of gene *j* with gene *i*. If *i* ≠ *j*, then 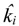 denotes the estimated basal level of the *i-*th gene. *n*(t) denotes the model residual and measurement noise. The steady state *x_s_* of *x*(*t*) is obtained as *t*→∞:

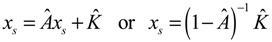
(2.8)


To simplify analysis of “robustness” of the steady state (phenotype), the origin of the dynamic system is shifted to the steady state *x_s_*, that is, *x*(*t*) = 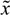
(*t*) + *x_s_*. This shift allows the following shifted dynamic system to be achieved by subtracting Equation (2.7) from Equation (2.8) [34]:

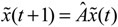
(2.9)


Therefore, the robustness of the phenotype (*i.e.*, the steady state) of the gene network becomes the robustness of the shifted gene network in Equation (2.9) at the origin 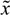
(*t*) ≡ 0. In the following, its network robustness (tolerance to intrinsic network perturbation) is tested. Suppose the linear GRN suffers from intrinsic molecular perturbations mainly due to process noise, thermal fluctuation, or genetic mutations. The interactive matrix *Â* is then perturbed as *Â*(1 + *η*), where *η* denotes the ratio of intrinsic perturbation. That is, the corresponding additional system perturbation is Δ*Â = ηÂ* [80]. A GRN with intrinsic perturbation can then be represented by

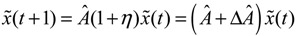
(2.10)


Because quadratic stability with the Lyapunov energy-like function *V*(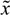) = *
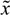
^T^*(*t*)*P
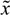
*(*t*) > 0, with *V*(0) = 0 for a positive symmetric matrix, *P* = *P^T^* > 0. The perturbative GRN in Equation (2.10) is robustly stable if Δ*V*(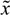) =* V*(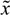
(*t+*1)) − *V*(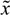
(*t*)) ≤ 0, *i.e.*, the energy of the gene network is not increased by intrinsic perturbations [79]. Based on this idea of robust stability, if the following inequality has a positive definite solution *P = P^T^ >* 0 [34],

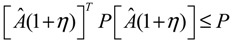
(2.11)
then the perturbative gene network is robustly stable or the phenotype of the gene network is maintained under parametric perturbation ratio *η*. The network robustness *η*° of the perturbative gene network is equal to the tolerance of the largest perturbation that does not violate network stability or still maintains the phenotype of the perturbative gene network:

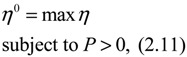
(2.12)


**Figure 4:**
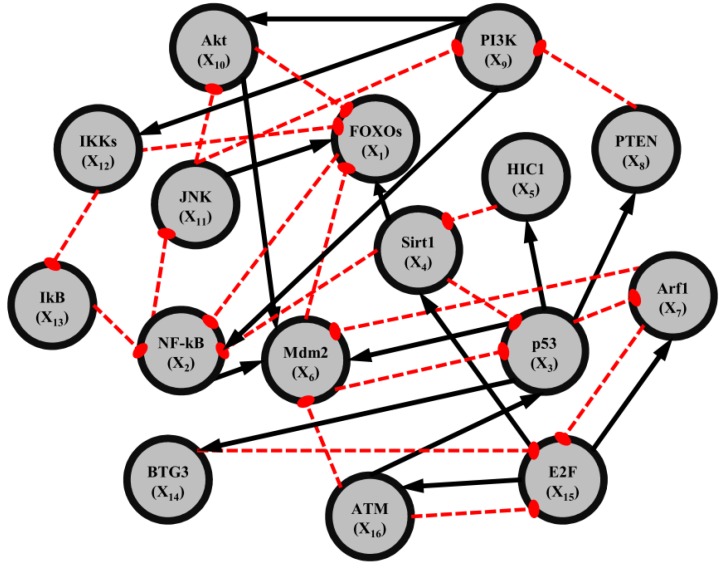
Multiple loops of a gene regulatory network associated with aging-related pathophyiological phenotypes. This aging-related gene regulatory network includes 16 genes: *FOXO_s_*, *NF-kB*, *P53*, *SIRT1*, *HIC1*, *Mdm2*, *Arf1*, *PTEN*, *P13K*, *Akt*, *JNK*, *IKK*, *IkB*, *BTG3*, *E3F1*, and *ATM*. Dashed red lines and black arrows indicate negative and positive parameters of regulated interaction, respectively.

That is, the network robustness is the maximum perturbation ratio *η*° tolerated by the GRN such that network stability (or phenotype) is still maintained. The constrained optimization problem in (2.12) can be solved by increasing *η* until no positive solution *P* exists in Equation (2.11) up to the highest value possible without violating the robust stability in Equation (2.11). A positive definite solution *P* > 0 in Equation (2.11) can be easily obtained by using the linear matrix inequality (LMI) Toolbox of Matlab. This network robustness method has been used to measure the relative network robustness of multiple loops of a gene regulatory network associated with aging-related pathophysiological phenotypes by using previously reported microarray data. It profiles the effects of aging on gene expression in the thymus, spinal cord, and eye tissues in mice [34]. This aging-related GRN includes 16 genes (Figure 4). The relative robustness *η*° toward normalized perturbation for GRNs of young, aged, and calorie-restriction (CR) groups is shown in Table 1. 

After the network robustness of aging-related GRNs is measured, the response abilities of genes to external stimuli are examined. Assuming that the GRN responds to external stimuli *U*(*t*), including upstream regulatory signals and external signals outside the network (e.g., hormones, carcinogens, oxidative stress, or ambient biomedical molecules) that could produce an effect on the gene network, the dynamic Equation (2.9) could be modified as follows:

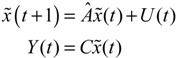
(2.13)
where *U*(*t*) = [*u*_1_(*t*) ⋯ *u_N_*(*t*)]*^T^* represents external stimuli and *Y*(*t*) denotes the output signal response of specific genes of interest. For example, if the output signal response of the gene *i* to external stimuli *U*(*t*) is to be analyzed, then *C* = diag(0, 0, ..., 0, 1, 0, 0, ..., 0), *i.e.*, all elements of *C* are zero except for a single element at the *i-*th diagonal component. For example, *C* = diag(0, 0, 1, 0, ..., 0) describes the gene response of the P53 gene. If the network response ability of the entire GRN to external stimuli is analyzed, then *C = I* (identity matrix).

The effect of input signals *U*(*t*) on output signal *Y*(*t*) is less than or equal to a positive value *δ*, if the following inequality holds [79]

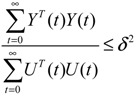
(2.14)
*δ* denotes the upper bound of the effect of *U*(*t*) on *Y*(*t*) for all bounded input signals *U*(*t*). *δ*°, the smallest upper bound of *δ* in (2.14), is called the “gene response ability (or sensitivity)” of the GRN. *δ*° permits us to obtain a more systematic insight into the ability of gene response to external stimuli for individual genes or the entire GRN. From the system gain result in [79], the network response of the dynamic GRN has an upper bound *δ* in (2.14), if there exists a positive definite *P = P^T^ >* 0 solution to the following LMI [34]:

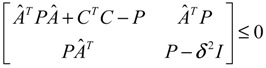
(2.15)


That is, if the above LMI holds for some *P > 0*, then the effect of *U*(*t*) on *Y*(*t*) must be less than or equal to *δ*, and Equation (2.14) holds. The response ability (minimal *δ*) of the GRN to external stimuli can be obtained by solving the following constrained optimization problem:

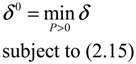
(2.16)


This can be solved by decreasing the upper bound *δ* in Equation (2.14) until no *P* > 0 exists in Equation (2.15) by using Matlab LMI Toolbox. By following Equation (2.16), the network response abilities of aging-related GRNs in Figure 4 can be measured and compared at different life stages. Results are shown in Table 1. 

Table 1 shows that the “aged” group has higher network robustness but lower network response ability. In order to tolerate the intrinsic parameter fluctuations accumulated due to genetic mutation, the elderly GRN becomes more robust than the young GRN. Robust GRNs are less responsive to external stimuli; consequently, the network protective ability against external stimuli decreases in the older gene networks. However, a more robust GRN may harbor more accumulated genetic mutations, which through random drift might provide more evolutionary paths to other phenotypes of gene network and thus lead to some aging-related chronic diseases like cancers, metabolic disorders, and arthritis. Table 1 also shows that the “young” network groups are less robust, with greater response to external stimuli. These GRNs are therefore also less robust to intrinsic perturbations and elicit a strong response toward external stimuli. This might imply that some gene expressions such networks could be easily reprogrammed to mediate downstream genes or regulators for further reactions. It also allows for modulation of gene expression in response to external stimuli, such as exposure to oxidative stress, carcinogens, and pro-inflammation molecules. These observations suggest that the gene regulatory network at the earlier stage of life or under conditions of CR may opportunely have protective adaptations to maintain intact regulatory structures and homeostasis of cellular functions. The purpose of the above analysis is to gain a greater understanding of the systematic protective and/or defensive mechanisms inherent to aging-related GRNs. The proposed robustness measurement methods may be used for future studies of GRNs involved in various biological processes and may have potential applications in gene therapy and drug target selection. It was also found that the network robustness of cancer cells is higher than that of normal cells and that the reverse is true for network response ability [33,34].

**Table 1:** The network robustness (*η*°) and network response ability (*δ*°) of a gene regulatory network with 16 aging-related genes (Figure 4) across different tissues at young, aged, and calorie-restrictive (CR) stages.

Tissue	Young	Aged	CR
Thymas			
*η*°	0.2310	0.3750	0.2050
*δ*°	1.1653	0.9463	1.2279
Spinal Cord			
*η*°	0.2410	0.6270	0.1400
*δ*°	1.1630	0.9367	1.2645
Eye			
*η*°	0.1600	0.2910	
*δ*°	1.2376	0.8798	

### 2.5. Network-Based Biomarker Determination via Sample Microarray Data Using a Systems Biology Approach

Biomarkers are used either in diagnostic evaluation to determine the health of a patient with or without a disease, or as a prognostic indicator to determine a patient’s prognosis. Present biomarker identification methods, which strictly use gene expression profiles, cannot show how the different genes within the biomarker gene set are related to each other; that is, biomarkers are not identified from a systems biology perspective. Furthermore, the gene lists obtained for similarly diagnosed patients by different research groups differ widely and share few common genes. Here, a systems biology approach is introduced for the integration of microarray data and PPI information to develop a network-based biomarker for systematic investigation into the network mechanism of lung carcinogenesis and the diagnosis of lung cancer [70]. The network-based biomarker consists of two protein association networks constructed from cancer and noncancer samples. The proposed method can be widely applied to determining network-based biomarkers for other diseases. The overall flowchart of the proposed network-based biomarker approach is shown in Figure S2 [71]. First, PPI information and microarray sample data for smokers with and without cancer are used to construct potential PPI networks for cancer and noncancer samples. Since data for both samples are limited, the number of proteins used in potential PPI network construction is also restricted: to avoid overfitting in network construction, the maximum degree of proteins in the potential network are less than the number of samples, imposing a limit on the size of the potential network.

Using a simple regression model, the potential PPI network is then further validated by the sample microarray data to highlight independent protein associations of both samples relative to their respective data sets. For a target protein *i* in the potential PPI network, the protein is described using the following protein association model [71]:

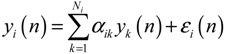
(2.17)
where *y_i_*(*n*) represents the gene expression level of the target protein *i* for the sample *n*, and *α_ik_* denotes the association ability between the target protein *y_i_*(*n*) and protein *y_k_*(*n*) for sample *n*. *N_i_* represents the number of proteins interacting with the target protein *i*; it can be obtained from the rough PPI network. *ε_i_*(*n*) denotes stochastic noise associated with other factors or model uncertainty. Equation (2.17) states that, biologically, the expression level of the target protein *i* is associated with the expression levels of interacting proteins.

The associated interaction parameter *α_ik_* in Equation (2.17) is identified through maximum likelihood estimation [77] on microarray data. AIC and Student’s t-test were employed for model order selection and for tests on the statistical significance of protein associations. Based on *α_ik_*, two matrices are established to represent the cancer protein association network (CPAN) and the non-cancer protein association network (NPAN) as follows [77]

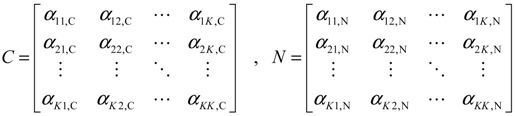
(2.18)
where *α_ij,C_* and *α_ij,N_* indicate the quantitative protein association ability between protein *i* and protein *j* for CPAN and NPAN, respectively, and *K* is the number of proteins in the protein association network. The resulting CPAN and NPAN constitute the network-based biomarker used for identifying significant proteins in lung carcinogenesis through the diagnostic evaluation

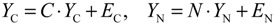
(2.19)
where *Y_C_* = [*y*_1,*C*(*n*)_ ⋯ *y_K_*_,*C*(*n*)_]*^T^*, *Y_N_* = [*y*_1,*N*(*n*)_ ⋯ *y_K_*_,*N*(*n*)_]*^T^* denotes the vectors of expression levels, and *E_C_* and *E_N_* indicate the noise vectors in cancer and non-cancer cases, respectively. A matrix indicating the difference between two protein association networks is defined as *C* − *N*, *i.e.*,

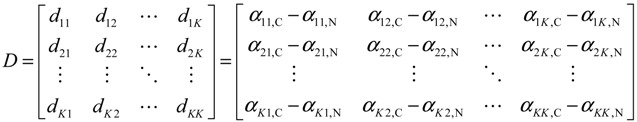
(2.20)
where *d_ij_* denotes the difference in protein association ability between CPAN and NPAN among proteins *i* and *j*. Using matrix D to represent the difference in network structure between CPAN and NPAN, a carcinogenesis relevance value (CRV) was derived to quantify the correlation of each protein significant to lung carcinogenesis. To identify significant proteins, two important issues are taken into consideration. First, the magnitude of the association ability *α_ij_* denotes the significance of association of one protein to another. A higher absolute value of *α_ij_* implies that the two proteins are more tightly associated. Second, if a protein plays a more crucial role in lung carcinogenesis, then the difference in association numbers linked to the protein for CPAN and NPAN would be larger. For example, if one protein shares a strong association with many proteins in CPAN, but has weaker associations (no protein) in NPAN, then the protein in question is more likely to be involved in lung carcinogenesis. As a result, CRV is determined based on the difference in protein association abilities using the following equation [70]:

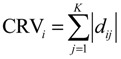
(2.21)


For the *i-*th protein in the network-based biomarker, the implication of Equation (2.21) is that the CRV quantifies the extent of protein associations that differentiate CPAN from NPAN.

**Figure 5:**
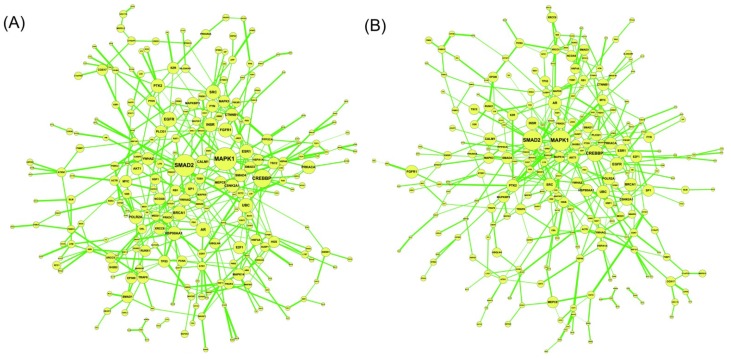
The constructed network-based biomarker. (**A**) Cancer protein association network (CPAN) obtained from *C* in (2.18) by maximum likelihood estimation, Akaike’s information criterion (AIC) selection, and Student’s t-test. (**B**) Non-cancer protein association network (NPAN) obtained from *N* in Equation (2.18) using the same criteria.

**Figure 6:**
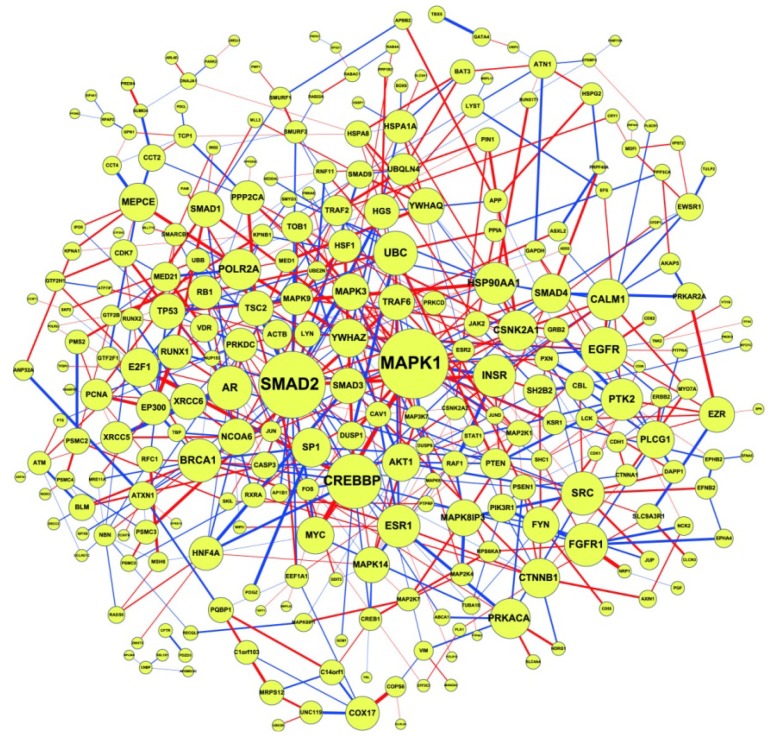
The difference between CPAN and NPAN obtained from Equation (2.20) for network-based biomarkers for lung cancer. The significance of proteins (indicated by circle size) to the network-based marker is dependent on their CRVs in Equation (2.21), which are listed in Table S1.

The above network-based biomarker approach is applied to the molecular investigation and diagnosis of lung cancer. The primary data set of GSE4115 (79 smokers with lung cancer and 73 smokers without lung cancer; obtained from the GEO database, http://www.ncbi.nlm.nih.gov/geo/) was used for construction of the network-based biomarker. The CPAN and NPAN in Equation (2.18), which consist of 399 and 393 protein associations respectively, constitute the network-based biomarker of lung cancer (Figure 5). The difference between CPAN and NPAN is shown in Figure 6. The CPAN indicates that 40 identified proteins play significant roles in lung carcinogenesis (Table S1).

### 2.6. On the Network Robustness and Filtering Ability versus Molecular Noise in GRNs Using a Stochastic System Approach

Molecular noise has been shown to play many roles in the cellular functions of GRNs, including noise-driven divergence of cell fates and population heterogeneity, noise-induced amplification of signals, generation of errors in DNA replication leading to mutation and evolution, and maintenance of the quantitative individuality of cells [50]. Other cellular processes influenced by noise include ion-channel gating, neural firing, developmental modules, cytoskeleton dynamics, and motors [50]. Phase variation in pathogenic bacteria, in which cells alternate randomly between expressing certain genes and silencing others, is thought to be a form of cultivated noise [25]. These molecular-level noisy phenomena are deeply rooted in the statistical mechanical behavior of so-called nanoscale chemical systems, where concentrations of reactive molecule species are extremely low and, consequently, fluctuations (noises) in the reaction rates are large [50]. Even though the molecular fluctuations leading to phase variation seem random in the individual, regulatory factors tune the variation to ensure mean levels of heterogeneity for the population, *i.e.*, the random molecular noises can be shown to be filtered or attenuated by the GRNs [25].

Since cellular molecular events are subject to significant thermal fluctuations and noisy processes with transcriptional control, alternative splicing, translation, diffusion, and chemical modification reactions, gene expression is best viewed as a stochastic process. Many observations suggest that molecular events underlying cellular physiology are subject to random fluctuations; these observations have led to the proposal of a stochastic model for gene expression and cellular functions [25]. Noise filtering can be considered from a signal processing perspective [74]. From this perspective, a pathway is viewed as an analog filter in terms of its frequency response. In terms of signal processing, these cellular pathways function as biological filters that transduce molecular signals and filter molecular noise [25]. 

**Figure 7:**
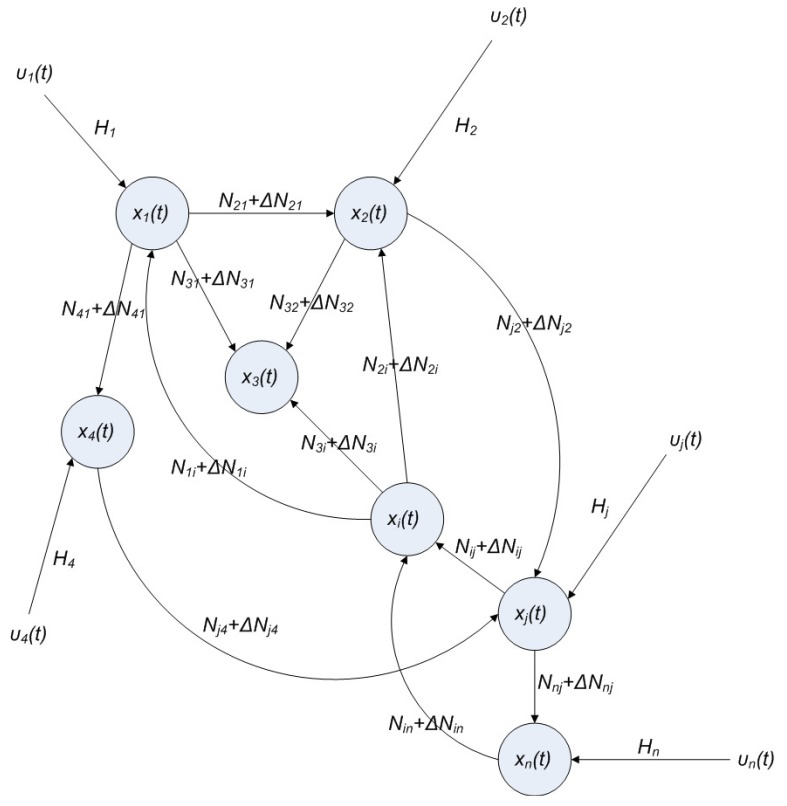
The linear *n* genes GRN with interaction *N_ij_*, intrinsic fluctuation Δ*N_ij_*, gene expression *x_i_*(*t*), and extrinsic fluctuation *υ_i_*(*t*).

For the convenience of illustration, the following linear biochemical dynamics of *n* genes GRN in Figure 7 [25] is simply considered initially:

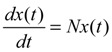
(2.22)
where the concentration vector *x*(*t*) and stoichiometric matrix *N* are given by

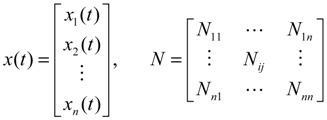

where *x_i_*(*t*) denotes the concentration of the *i-*th gene, and *N_ij_* denotes the interaction between genes *j* and *i*.

Suppose the linear GRN suffers intrinsic molecular fluctuations so that the stoichiometric matrix *N* is perturbed as *N +* Δ*N*,

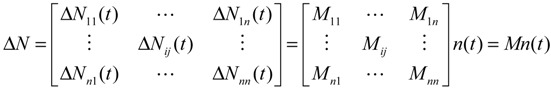

where Δ*N_ij_* denotes the random parametric fluctuation of *N_ij_*; *M_ij_* denotes the deterministic part (amplitude) of fluctuation; and *n*(*t*) is white Gaussian noise with zero mean and unit variance, and denotes the stochastic part of fluctuation,* i.e.*, the stochastic part of fluctuation is absorbed to *n*(*t*).

The GRN with random parametric fluctuation can then be represented by

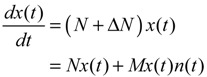
(2.23)


For the convenience of analysis by the stochastic process, the above stochastic GRN can be represented as follows [74,75]:

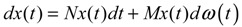
(2.24)
where *dω*(*t*) *= n*(*t*)*dt*. *ω*(*t*) is called the stochastic Wiener process or Brownian motion [73].

The stochastic dynamic equations of GRNs are always nonlinear. In order to meet the nonlinear stochastic regulatory networks, Equation (2.23) should be generalized as the following Langevin equation [25]

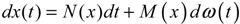
(2.25)
where *N*(*x*) denotes the nonlinear interaction equation of the nonlinear GRN, and *M*(*x*)*dω*(*t*) is due to nonlinear intrinsic random fluctuation.

Based on stochastic Lyapunov theory, let *V*(*x*) > 0 with *V*(0) = 0 denote the Lyapunov power-like function, then the stochastic GRN in Equation (2.23) or (2.24) is stochastically stable if E[*dV*(*x*(*t*))/*dt*] ≤ 0 [73]. With the choice of *V*(*x*) = *x^T^*(*t*)*Px*(*t*) for some positive definite matrix *P*, the following result is derived.

**Proposition 1** [25]:

The linear GRN with stochastic perturbation in Equation (2.23) or (2.24) is stochastically stable if the following LMI

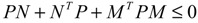
(2.26)
has a symmetric positive definite solution *P >* 0, *i.e.*, the phenotype of the linear stochastic GRN is maintained under intrinsic stochastic fluctuation.

**Remark 1**: (i) In the intrinsic noise-free case, the stable condition in (2.26) is reduced to *PN* + *N^T^P* ≤ 0, *i.e.*, the eigenvalues of system matrix *N* should be on the left-hand side of the complex domain. Obviously, if the LMI in Equation (2.26) has a positive solution *P >* 0, then the eigenvalues of *N* should be located on the far left-hand side of the complex domain with more negative real values in order to overcome the additional term *M^T^PM* due to intrinsic random noise. (ii) If some eigenvalues of system interaction matrix *N* are near the *jω* axis, then intrinsic random molecular fluctuations across the *jω* axis perturb these modes more easily such that the linear GRN becomes unstable. The LMI in Equation (2.26) can be rearranged to

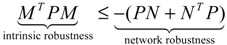
(2.27)
that is, −(*PN* + *N^T^P*) in Equation (2.27) can be taken as a measure of network robustness, and *M^T^PM* due to the random parametric fluctuation can be taken as a measure of intrinsic robustness. The physical interpretation of Equation (2.27) is that if the network robustness can confer enough intrinsic robustness for tolerating intrinsic random parameter fluctuation, then the phenotype of the GRN is maintained.

For the nonlinear stochastic GRN in Equation (2.25), the following result is also derived.

**Proposition 2** [25]:

The nonlinear stochastic GRN in Equation (2.25) is still stochastically stable if the following Hamilton–Jacobi inequality (HJI) has a positive Lyapunov solution *V*(*x*) > 0 with *V*(*0*) = 0

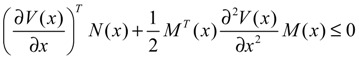
(2.28)
That is, the phenotype of the nonlinear stochastic GRN in Equation (2.25) is maintained under intrinsic stochastic fluctuation. Similarly, the HJI in Equation (2.28) can be rearranged as the following phenotype robustness criterion:

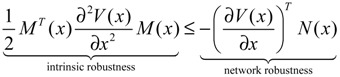
(2.29)
That is, if the network robustness of the nonlinear stochastic GRN in Equation (2.25) can confer enough intrinsic robustness to tolerate intrinsic stochastic fluctuation, then the phenotype of the GRN is maintained.

After the robust stability of GRN is guaranteed under intrinsic biochemical stochastic fluctuation, the effect of environmental random molecular noises on the GRN may be discussed. If the linear GRN in Equation (2.24) also suffers from environmental molecular noises *ν*(*t*) = [*ν*_1_(*t*) ⋯ *v_n_*(*t*)]*^T^* outside the network (see Figure 7), then

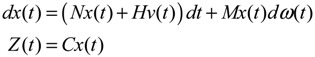
(2.30)
where *H* is a coupling matrix denoting the influence of environmental molecular signals *ν*(*t*) on the GRN. *Z*(*t*) denotes the concentration of specific genes of interest. For example, if we want to examine the effect of molecular noises of *ω*(*t*) and *ν*(*t*) on gene *i* (*i.e.*, *x_i_*(*t*)), then we let *C* = diag(0…010…0). That is, every element of *C* is zero except for the *i-*th element. To investigate the effect of molecular noises on the whole GRN, then *C = I*, the identity matrix. The positive value *ρ* in the following inequality is then called the effect of environmental noises (or signals) on *Z*(*t*) in the stochastic GRN in Equation (2.30) with *x*(0) = 0

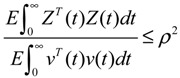
(2.31)
That is, *ρ* is the upper bound of the effect of all environmental molecular signals or noises *v*(*t*) on the GRN. It is called the response level of the GRN.

From this is derived the following response level result of the GRN in Equation (2.30).

**Proposition 3** [25]:

The response level *ρ* of the linear stochastic GRN in Equation (2.30) is guaranteed if the following matrix inequality has a positive solution *P* > 0

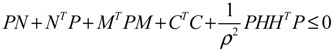
(2.32)
By Shur complement [79], the above inequality is equivalent to the following LMI:

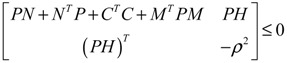
(2.33)
That is, if the above LMI has a positive solution *P* > 0, then the linear stochastic GRN in Equation (2.30) is robustly stable under random intrinsic molecular fluctuation and has a response level *ρ* to environmental molecular noises.

The optimal response level *ρ*_0_ of the linear stochastic GRN (Equation (2.30)) can be obtained by solving the following constrained optimization

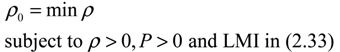
(2.34)


**Remark 2**: (i) If *ρ*_0_ < 1, then environmental molecular noises *υ*(*t*) are attenuated by the GRN and *ρ*_0_ is called the filtering ability of the GRN, *i.e.*, the GRN is less sensitive to environmental noises. If *ρ*_0_ > 1, then environmental molecular noises are amplified by the GRN, *i.e.*, the GRN is more sensitive to environmental noises. (ii) Substituting *ρ*_0_ for *ρ* in Equation (2.32) and rearranging, the following phenotype robustness criterion of the stochastic GRN is derived

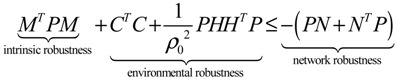
(2.35)


The phenotype robustness criterion in (2.35) for the stochastic GRN in (2.30) can be denoted as “intrinsic robustness + environmental robustness ≤ network robustness”. In other words, if the sum of intrinsic robustness and environmental robustness is less than network robustness, then the phenotype of the stochastic GRN remains robust under the influence of stochastic intrinsic fluctuation and environmental noises. In order to maintain the phenotype robustness criterion, GRNs need negative feedback loops to improve the network robustness on the right-hand side of Equation (2.35). Parallel loops and modular and redundant structures are also required to reduce the effect of intrinsic parameter variations on the GRN and to resist environmental disturbance, *i.e.*, to provide more intrinsic robustness and environmental robustness on the left hand side of Equation (2.35). This is why feedback loops, parallel loops, and modular and redundant structures are abundant in GRNs as they contribute to phenotype robustness and favor natural selection. (iii) If the network robustness in Equation (2.35) is not large enough to confer intrinsic robustness and environmental robustness simultaneously to maintain the phenotype of the GRN, some negative feedback gene loops are implemented as follows:

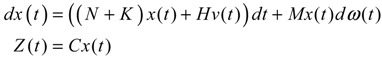
(2.36)
where *K* denotes specific negative feedback loops that improve the network robustness of the stochastic GRN. In this situation, the phenotype criterion in Equation (2.35) is modified as

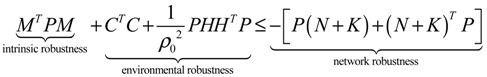
(2.37)


It can be seen from the phenotype robustness criterion in Equation (2.37) that an adequate *K* can improve the network robustness of the stochastic GRN such that it better tolerates intrinsic stochastic fluctuation and is less responsive to environmental noise (i.e. smaller *ρ*_0_).

Similarly, the nonlinear stochastic GRN under environmental molecular noises (Equation (2.25)) should be modified as follows:

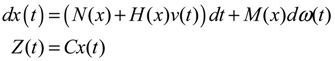
(2.38)


The phenotype robustness criterion in Equation (2.37) is then modified as follows [25]:

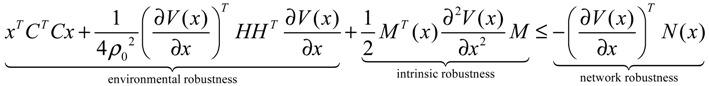
(2.39)


In general, it is very difficult to solve the HJI in Equations (2.29) and (2.39). Global linearization techniques [79,81] or T-S fuzzy methods [82,83] can be employed to interpolate several local linearized systems to approximate the nonlinear stochastic system in Equation (2.38) as follows:

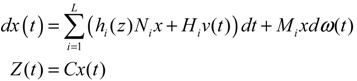
(2.40)
where *h_i_*(*z*), *i* = 1,⋯, *L* denotes the interpolation functions of global linearization or fuzzy bases with *h_i_*(*z*) ≥ 0, 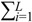 *h_i_*(*z*) = 1, and *dx* = (*N_i_x* + *H_i_v*)*dt* + *M_i_xdω* is the *i-*th local linearized GRN.

In this situation, the network robustness criterion in Equation (2.39) can be modified accordingly [25]

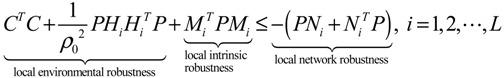
(2.41)


From the phenotype robustness criterion in Equation (2.41), it is seen that if local network robustness is large enough for every local linearized GRN to simultaneously provide enough local intrinsic robustness for tolerating local random parameter fluctuations and enough local environmental robustness for resisting local environmental disturbance, then the phenotype of nonlinear stochastic GRN is maintained in spite of intrinsic stochastic parameter fluctuations and environmental molecular noises. If the phenotype robustness criterion in Equation (2.41) cannot be maintained, then negative feedback loops can be engineered to improve network robustness. These feedback loops may have potential applications for some types of therapy and drug target selection. 

Systematic methodologies for the analysis of system characteristics of GRNs and PPINs detailed above are useful for the systematic designs of systems synthetic biology discussed in the following section.

## 3. Systems Synthetic Biology

Synthetic biology can be expected to have important applications in biotechnology and medicine, and to contribute significantly to a better understanding of the functioning of complex biological systems. Synthetic biology is concerned with the engineering of biological systems that fulfill a particular purpose. This is achieved by transformative innovation that makes it possible to build living machines from off-the-shelf chemical ingredients, employing many of the same strategies that electrical engineers use to make computer chips. The main goal of this nascent discipline is the design and construction of biological systems with desired behaviors [45,46,47,48,49]. Synthetic biology envisions the redesign of natural biological systems based on a set of powerful biomolecular techniques for the automated synthesis of DNA molecules and their assembly into genes and microbial genomes, for greater efficiency, as well as for the construction of functional “genetic circuits” and metabolic pathways for practical purposes [49]. The construction of GRNs has recently demonstrated the feasibility of synthetic biology [84,85,86,87]. The design of robust gene networks still presents a difficult challenge, and most newly designed gene networks cannot function properly. Such design failures are mainly due to both intrinsic perturbations such as gene expression noises, splicing, mutation, evolutionary changes, and environmental disturbances such as changing extracellular environments [52,53]. Designing robust synthetic gene networks that can tolerate intrinsic parameter perturbations, attenuate extrinsic disturbances, and function properly in a host cell is therefore an important topic in synthetic biology [55].

### 3.1. Design of Specifications-Based Systems Synthetic Biology

Analysis of the dynamic properties of gene networks has been previously implemented using sensitivity analysis, either by qualitative simulation of coarse-grained models or by extensive numerical simulations of nonlinear differential equation models or stochastic dynamic models [56]. For applications in systems synthetic biology, however, these approaches are not satisfactory since local sensitivity analysis can provide only a partial description of all possible behaviors of a nonlinear gene network. In particular, it cannot guarantee that a synthetic network behaves as expected for all uncertain initial conditions, external disturbances, and parameter variations in a given range. Recently, Kuepfer *et al.* developed an approach based on semidefinite programming for partitioning parameter spaces of polynomial differential equation models into “feasible” and “infeasible” regions [68]. In this approach, “feasible” simply refers to the existence of a desired steady state of the synthetic network. Another approach using robustness analysis and tuning of synthetic networks was proposed by Batt *et al.* to provide a means to assess the robustness of a synthetic gene network with respect to parameter variations [51]. This approach allows searching for parameter sets for which a given property is satisfied, using a publicly available tool called RoVerGeNe. Several gene circuit design methods have recently been introduced insert or delete specific circuits in an existing gene network to modify its structure toward improved robust stability and filtering ability [38]. Robust synthetic gene network design, however, is a topic in itself, necessitating the design of a completely synthetic network with enough robust stability toward parameter fluctuations and with enough noise filtering ability to resist external disturbances, which allows it to function properly in a host cell. 

**Figure 8:**
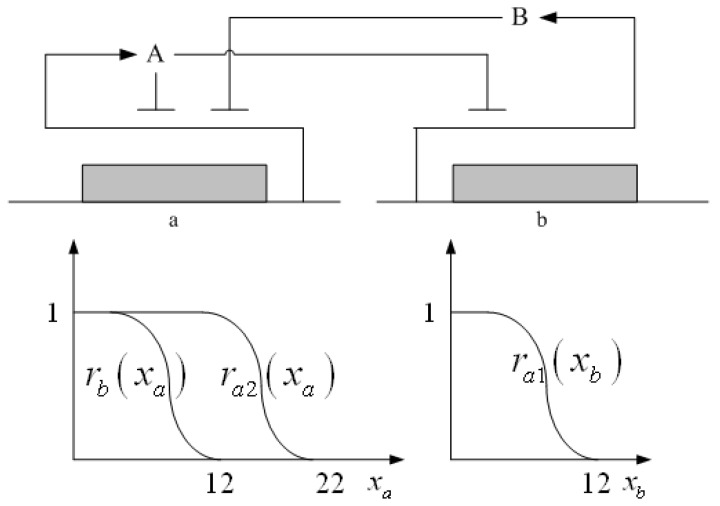
A simple two-gene cross-inhibition network. The network’s regulation functions are given in Equations (3.1) and (3.2).

A robust synthetic biological design incorporating molecular noises has been developed based on stochastic game theory [52]. However, the intrinsic parameter fluctuations of synthetic gene networks have not been considered in the design procedure. In a previous study [53] some system design specifications (engineering designs) had been provided by users so that the designer must engineer an artificial gene network to meet these design specifications. For the convenience of problem description, a simple design example is provided to give an overview of design problems for robust synthetic gene networks. A more general problem scenario is treated in preparation for this overview. First, consider the cross-inhibition network shown in Figure 8. This network is synthesized with two genes (a, b) that code for two repressor proteins (A, B). More specifically, protein A represses the expression of gene b, and, at higher concentration, the expression of its own gene. Protein degradation is not regulated. This synthetic system can be modeled by the following synthetic equations:

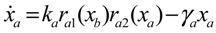
(3.1)

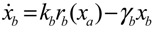
(3.2)


The state variables *x_a_* and *x_b_* denote the concentrations of proteins A and B, respectively. *k* and *γ* are the kinetic parameter and decay rate, respectively. *r* is the regulation function capturing the regulator effect of a transcriptional protein on gene expression, and has a smooth sigmoidal form (e.g., Hill function) [88].

The simple cross-inhibition network in (3.1) and (3.2) can be represented by the following stoichiometric matrix equation [7]

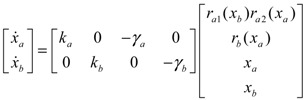
(3.3)


However, the stoichiometric matrix *in vivo* suffers from intrinsic parameter perturbations because of gene expression noises, splicing, mutation, evolutionary change, *etc.*, as in [52]

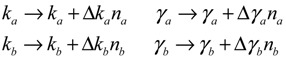
(3.4)
where Δ*k_i_* and Δ*γ_i_* denote the amplitudes of fluctuations of the stochastic parameters and decay rates, respectively; and *n_i_* is a random white noise with zero mean and unit variance.

Suppose the synthetic gene network also suffers from environmental disturbances because of changing extracellular environments and interactions with the cellular context in its host cell. The stochastic gene network can be then represented as

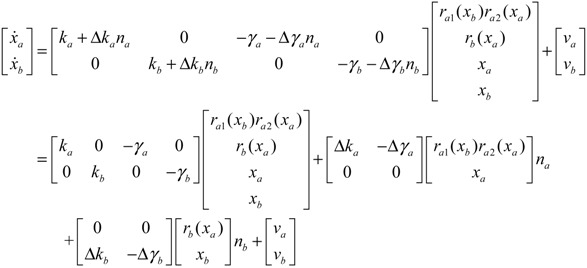
(3.5)
where *x* = [*x_a_ x_b_*]*^T^* and *ν* = [*ν_a_ ν_b_*]*^T^* denote the state vector and environmental disturbance of the synthetic gene network in the host cell, respectively. These intrinsic parameter fluctuations and environmental disturbances may cause the engineered synthetic gene network to be dysfunctional in the host cell.

If a synthetic gene network consists of *n* genes, then the stochastic gene network of Equation (3.5) in the host cell can be extended according to the following n-gene network dynamics [52]

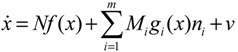
(3.6)
where the state vector *x* = [*x*_1_⋯*x_n_*]*^T^* denotes the concentrations of proteins in the synthetic gene network, *N* denotes the corresponding stoichiometric matrix of the n-gene network, *M_i_*'; *i* = 1,..., *m* denotes fluctuation matrices associated with independent random noise sources *n_i_*, *i* = 1,..., *m*; and the elements of *M_i_* denote the standard deviations of the corresponding parameter fluctuations. *ν* = [*ν*_1_⋯*ν_n_*]*^T^* denotes the vector of external disturbances. This stochastic system is used to mimic the realistic dynamic behavior of a synthetic gene network of *n* genes in the host cell. As the network is subject to intrinsic parameter fluctuations and environmental disturbances in the context of the host cell, a robust synthetic gene network should be designed with the ability to not only tolerate parameter fluctuations and attenuate external disturbances, but also to achieve desired steady-state behaviors.

For convenience of analysis and design, the stochastic dynamic in Equation (3.6) of a synthetic gene network *in vivo* can be represented by the following Ito stochastic differential equation [73,74]

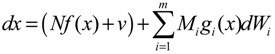
(3.7)
where *W_i_*(*t*) is a standard Wiener process with *dW_i_*(*t*) = *n_i_dt*.

To ensure correct and efficient operation of the gene network, several systematic design specifications should be imposed on it from the systematic engineering point of view.

(i) The kinetic parameters and the decay rates in stoichiometric matrix should be chosen from a biologically feasible range

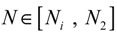



(ii) The network should tolerate the stochastic kinetic parameter and decay rate fluctuations with prescribed standard deviations in *M_i_* in the state-dependent noise term

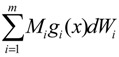



(iii) The following desired steady state should be achieved

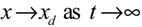



(iv) The following prescribed attenuation level *ρ* of environmental disturbance should be achieved (*i.e.*, the *H*_∞_ filtering ability)

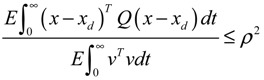
(3.8)
for all bounded environmental disturbances *v*(*t*), where *Q* ≥ *0* is a symmetric weighting matrix and *ρ* is a prescribed attenuation level less than 1. That is, the effect of environmental disturbance *v* on the regulation error *x* − *x_d_* should be less than the attenuation level *ρ* from the average energy perspective. 

In order to achieve the desired steady state *x_d_* and for convenience of design, the origin of the nonlinear stochastic system in Equation (3.7) should be shifted to *x_d_*. Stabilizing the shifted nonlinear stochastic system at the origin would then also achieve *x_d_*, simplifying the design procedure. Let 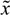 *= x* – *x_d_*. The following shifted stochastic synthetic genetic system is then derived [79]:

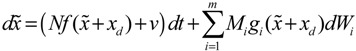
(3.9)
That is, the origin 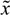 = 0 of the stochastic system in Equation (3.9) is at the desired steady state *x_d_* of the original stochastic system in Equation (3.7). *N* ∈ [*N*_1_,*N*_2_] is then specified to tolerate the stochastic parameter fluctuation 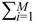 *M_i_g_i_*(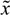 + *x_d_*)*dW_i_* and efficiently attenuate the environmental disturbance *v* to the prescribed level

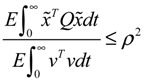
(3.10)


From the stochastic synthetic gene network Equation (3.9)* in vivo*, we obtain the following result.

**Proposition 4** [53]:

If design kinetic parameters and decay rates in *N* ∈ [*N*_1_,*N*_2_] are chosen such that the following HJI has a positive solution *V*(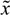) > 0

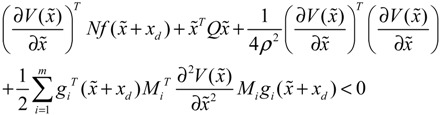
(3.11)
then (a) the stochastic gene network in Equation (3.9) can achieve both the robust stabilization necessary to tolerate the intrinsic stochastic parameter perturbations and the prescribed attenuation level *ρ* of environmental disturbances, *i.e.*, design specifications (i), (ii), and (iv) are satisfied. (b) If the stochastic gene network is free of environmental disturbances (*v*(*t*)* =* 0), then 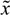
→0 or *x*→*x_d_* in probability, *i.e.*, design specification (iii) is achieved.

It is generally very difficult to specify *N* ∈ [*N*_1_,*N*_2_] to solve HJI in Equation (3.11) for *V*(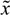) > 0 using a systematic method. If all global linearizations are bound by a polytope consisting of *M* vertices

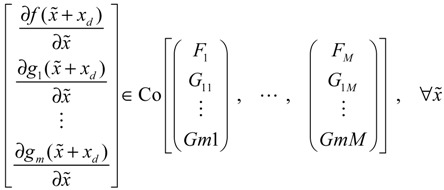
(3.12)
where Co denotes the convex hull of a polytope with *M* vertices defined in Equation (3.12), then through the global linearization method [79,81], the state trajectories 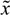
(*t*) of the shifted gene network in Equation (3.9) can be represented by the convex combination of *M* linearized gene networks as

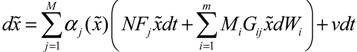
(3.13)
where the interpolation function *α_j_*(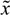) satisfies 0 ≤ *α_j_*(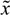) ≤ 1 and ∑*i* = 1*M* *α_j_*(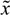) = 1. The trajectory of the nonlinear stochastic synthetic gene network in Equation (3.9) could thus be represented by the interpolated synthetic gene network in Equation (3.13). This yields the following result.

**Proposition 5** [53]:

Assuming that design kinetic parameters and decay rates in *N* ∈ [*N*_1_, *N*_2_] are chosen such that the following *M* LMIs have a common symmetric positive definite solution *P* > *0*

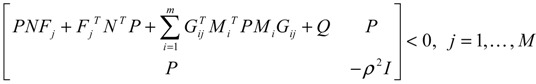
(3.14)
then there are two results: (a) The stochastic synthetic gene network in Equation (3.7) is robustly stable toward intrinsic stochastic parameter perturbations and achieves a prescribed attenuation level *ρ* of environmental disturbances. That is, design specifications (i), (ii) and (iv) are satisfied. (b) If the synthetic gene network is free of environmental disturbance (*v*(*t*)* =* 0), then the synthetic gene network in Equation (3.7) may asymptotically converge to the desired steady state. (An *in silico* design example is shown in supplementary example 1.)

**Remark 3**: (i) Gene circuit design can now be implemented using recombination technology to insert or delete TF binding sites in the promoter region of a regulated gene with the aim of increasing or decreasing the value of the kinetic parameter *κ_i_* (*i.e.*, different levels of affinity) of the regulated gene [89]. By inserting strong or weak binding sites, large or small values can be obtained. For example, the binding site of *κ_i_* = 1 is 10 times larger than that of *κ_i_* = 0.1 at the promoter region of target gene *i*. Changes to the decay rate *γ_i_* can be implemented by shortening the 3' polyadenylate tail (referred to as deadenylation), which primarily triggers decapping, resulting in 5' to 3' exonucleolysis. Alternatively, removal of the 3' polyadenylate tail can increase *γ_i_* [53]. Therefore, by shortening or elongating the gene’s 3' polyadenylate tail, we can increase or decrease the decay rate *γ_i_* of gene *i*. Directed evolution methods are also useful in changing the elasticity (kinetic property of *κ_i_*) and in designing biochemical circuits [53]. From a systems biology perspective, these advances in implementation techniques of *κ_i_* and *γ_i_* enable engineering of synthetic gene networks in the near future.

(ii) Because of the uncertain values of *v* and initial state 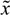
(0) of the synthetic gene network (3.9) in the host cell, the following minimax design has also been considered to reset the uncertainty of *ν*(*t*) and 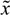
(0)* in vivo* [52]. *ν*(*t*) and 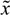
(0) are considered players maximizing their effects on regulation error 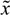
(*t*) when the kinetic parameters are specified as another player minimizing the effects of *v* and 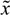
(0) on 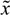
(*t*). 



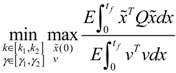
(3.15)


**Figure 9:**
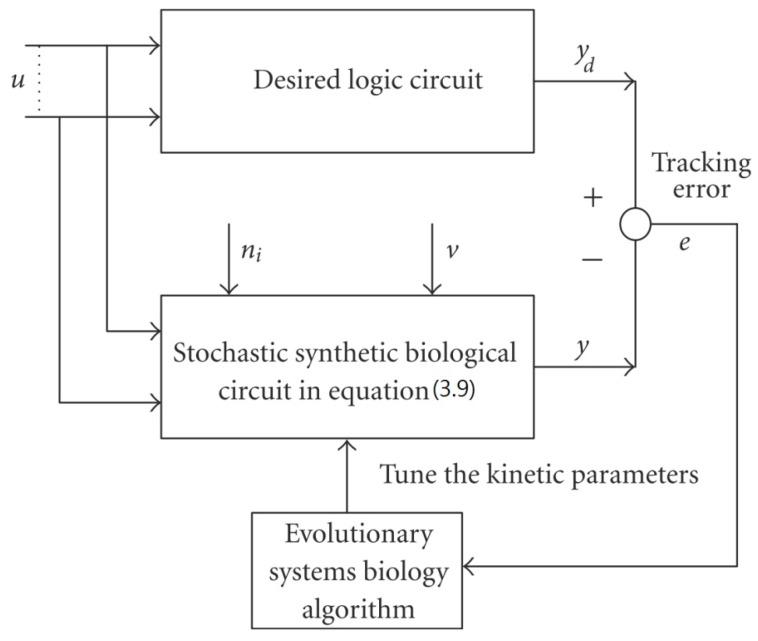
Block diagram of the optimal tracking scheme for synthetic biological circuit design using an evolutionary systems biology algorithm. Based on a network algorithm mimicking natural selection in an evolutionary process, the design parameters *k* of a synthetic biological circuit are tuned to minimize the tracking error between the desired logic circuit and the stochastic synthetic biological circuit, and to achieve the desired behavior tracking.

A robust synthetic gene network based on dynamic game theory and fuzzy interpolation of local linearized linear systems can thus be efficiently designed [52].

(iii) When the desired output of the synthetic gene network is not a steady state but a time-varying signal like a specific binary or periodic signal, and the network structure becomes complex, the above systematic analysis methods are difficult to apply. Because natural selection of traits suited for environmental change is an important evolutionary mechanism, kinetic parameters of synthetic gene networks can be tuned by genetic algorithms (GAs) or evolution algorithms (EAs) to optimally track desired biological functions [54,58]. Based on the evolutionary network algorithm, the kinetic parameters may be tuned to maximize the fitness to some desired phenotype selected by natural selection. Consider the optimal tracking design of a synthetic gene network by network evolution shown in Figure 9. Let the tracking error be defined as

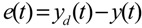



The design purpose is then to tune the design parameter *k_i_* using evolutionary (or genetic) algorithms such that the network can achieve the following optimal tracking

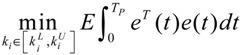
(3.16)
where *T_p_* denotes the present time. Let

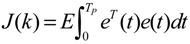
(3.17)


Let the fitness function *F*(*k*) be defined as

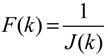
(3.18)


Adapting a parameter vector (chromosome) *k_i_* ∈ [*k_i_^L^*,*k_i_^U^*] by EA or GA to minimize *J*(*k*) for the desired network behavior tracking of the synthetic gene network is equivalent to maximizing the fitness function in Equation (3.18) to meet the natural selection. A robust biological network design with a desired output behavior *y_d_*(*t*) is therefore equivalent to as solution to the following fitness maximization problem using an evolutionary network method [54,58]

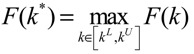
(3.19)


The evolutionary network algorithm (or evolutionary systems biology algorithm [58]) is employed to solve the above fitness maximization problem via genetic operators such as selection, crossover, and mutation. It mimics natural selection in an evolutionary process to tune the kinetic parameter network of the synthetic gene network to an environmental change. A GA-based algorithm with binary coding of the chromosome has been proposed for the design of robust synthetic genetic oscillators with prescribed amplitude, period, and phase [54]. The oscillator is intended to allow protein concentrations to track desired periodic reference signals under intrinsic and environmental noises. Based on an evolutionary systems biology algorithm that encodes each chromosome in a real-valued vector, the design parameters of target gene circuits can evolve to specific values in order to robustly track a desired biological function in spite of such interferences [58].

### 3.2. Robust Synthetic Gene Network Design via Library-Based Search Methods

Over the past decade, synthetic biology has made significant progress in designing biological parts. Even synthetic gene networks may have been constructed using a variety of biological components to achieve desired behaviors. A limitation on the development of complex synthetic gene networks intended to track specific reference trajectories is the lack of an efficient method for selecting suitable biological parts from libraries; experimental data in promoter libraries cannot be directly used for selecting adequate promoter parts. Current promoter libraries therefore need to be redefined based on promoter activity. This would allow development of library-based search methods.

**Figure 10:**
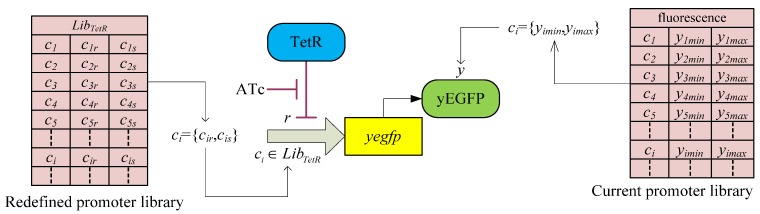
Single schematic diagram of the synthetic promoter-regulation gene circuit. The existing TetR-regulated promoter library contains the minimum and maximum values of fluorescence [*y_imin_*, *y_imax_*] corresponding to with and without TetR (repressor) binding. Based on the promoter regulation function (3.21) and these values, the promoter library is redefined for the design of synthetic gene networks (Table 2).

A dynamic model can be used in the indirect evaluation of the activities of promoter parts to help redefine existing promoter libraries. In the TetR-regulated promoter library shown in Figure 10, *x*(*c*, *t*) and *X*(*c*, *t*) respectively denote the concentrations of the mRNA and protein of gene *yegfp* (which is used to measure protein expression). The dynamic model of the promoter-regulation gene part is constructed with [56,57]

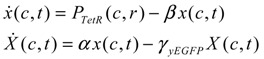
(3.20)
where *β* and *γ_yEGFP_* denote the degradation rates of mRNA and protein yEGFP, respectively, and *α* denotes the translation rate. The promoter regulation function *P_TetR_*(*c*,*r*), which is dependent on repressor activity *γ* and the choice of promoter *c*, has the form

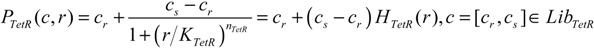
(3.21)
where the promoter *c* has two promoter activities *c_r_* and *c_s_* (the minimum and maximum values of the promoter regulation function *P_TetR_*(*c*, *r*), respectively) for the TetR-regulated promoter library *Lib_TetR_*. That is, *c* = (*c_r_*, *c_s_*) ∈ *Lib_TetR_*. *K_TetR_* and *n_TetR_* denote the TetR–DNA binding affinity and binding cooperativity of regulatory protein TetR and DNA, respectively. *H_TetR_*(*r*) is a Hill function capturing the effect of a regulatory protein. 

**Figure 11:**
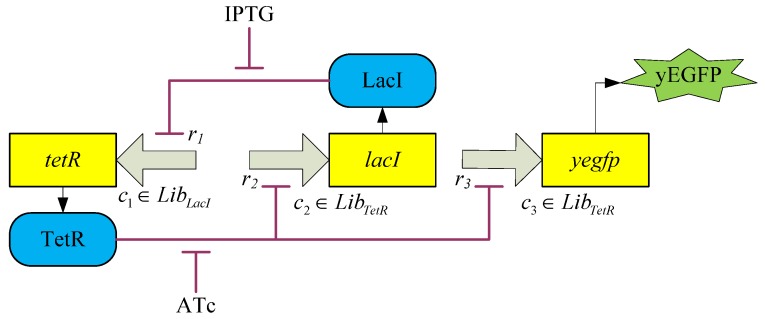
Synthetic gene circuit topology: simple toggle switch. The regulatory protein TetR, which is induced by ATc, inhibits the transcription of *lacI* by binding promoter *c*_2_. TetR also inhibits transcription of *yegfp* by binding promoter *c*_3_ to repress the expression of the fluorescent protein yEGFP. The protein LacI, which is induced by the inducer IPTG, inhibits the transcription of *tetR* by binding promoter *c*_1_. The gene circuit has two distinct stable states, and can reversibly switch between them by changing the inducers ATc and IPTG. If an adequate promoter set *c* = [*c*_1_, *c*_2_, *c*_3_] is selected from corresponding promoter libraries, then yEGFP can be used to track the desired behaviors generated by a reference model. In the reference model, *c*_1_ is selected from the LacI-regulated promoter library, and *c*_2_ and *c*_3_ are selected from the TetR-regulated promoter library in Table 2 (*i.e.*, *c*_1_ ∈ *Lib_LacI_*, *c*_2_, *c*_3_ ∈ *Lib_TetR_*).

Based on the estimated promoter activities *c* = (*c_r_*, *c_s_*) via maximum and minimum values of the steady state of protein expression data, some promoter libraries can be redefined in such a way that they can be efficiently selected from the design of the synthetic gene network (Table 2) [56,57]. Since a synthetic gene network always consists of a set of promoter-regulation gene circuits (Figure 10), the design of complex synthetic gene network addresses how to select a set of promoters from the corresponding promoter libraries that have promoter activities adequate for achieving the design specifications. A well-known gene circuit topology, the simple toggle switch, is shown for illustration purposes in Figure 11. The toggle switch has two distinct stable states and can be reversibly switched between them by changing the inducers ATc and IPTG. Let *x*_1_(*c*_1_, *t*), *x*_2_(*c*_2_, *t*), and *x*_3_(*c*_3_, *t*) denote the concentrations of mRNAs *tetR*, *lacI*, and *yegfp*, respectively; and let *X*_1_(*c*_1_, *t*), *X*_2_(*c*_2_, *t*), and *X*_3_(*c*_3_, *t*) denote the concentrations of proteins TetR, LacI, and yEGFP, respectively. The dynamic model of the toggle switch gene network in Figure 11 is then modeled as

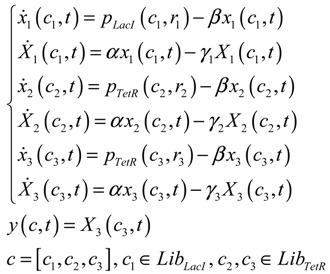
(3.22)


The promoter regulation functions *p_LacI_*(*c*_1_, *r*_1_), *p_TetR_*(*c*_2_, *r*_2_), and *p_TetR_*(*c*_3_, *r*_3_) are dependent on the selection of promoters *c*_1_, *c*_2_, and *c*_3_ from the corresponding promoter libraries in Table 2. The output of interest *y*(*c*, *t*) is dependent of the selected promoter set *c* = [*c*_1_, *c*_2_, *c*_3_] with adequate promoter activities from the corresponding promoter libraries in Table 2. This dynamic toggle switch gene network model consists of three interactive dynamic models of promoter-regulation gene parts, as shown in Equation (3.20).

**Table 2:** Redefined TetR- and LacI-regulated promoter libraries. The redefined TetR- and LacI-regulated promoter libraries (*i.e.*, *Lib_TetR_* and *Lib_LacI_*) comprise different promoters (*i.e.*, *T_k_* and *L_k_*, *k* = 0,…,20) with their corresponding activities of *c_s_* and *c_r_* obtained from previous libraries of experimental data.

TetR-regulated promoter library (Lib_TetR_)			LacI-regulated promoter library (Lib_LacI_)		
Promoter	Promoter activity		Promoter	Promoter activity	
	c_s_	c_r_		c_s_	c_r_
*T*_0_	2121	0.1724	*L*_0_	1657.5	0.3018
*T*_1_	1604	0.7576	*L*_1_	923.97	0.2567
*T*_2_	1376.6	0.1936	*L*_2_	860.87	0.2244
*T*_3_	1169.8	0.4672	*L*_3_	674.92	1.9189
*T*_4_	974.52	0.0753	*L*_4_	651.58	1.1680
*T*_5_	942.77	0.2281	*L*_5_	570.07	3.5062
*T*_6_	967.17	0.1493	*L*_6_	527.83	0.5497
*T*_7_	738.57	0.0702	*L*_7_	323.45	0.1248
*T*_8_	641.74	0.7135	*L*_8_	327.77	0.1772
*T*_9_	564.24	0.2620	*L*_9_	309.74	0.5439
*T*_10_	501.35	0.0756	*L*_10_	298.35	0.1146
*T*_11_	469.35	0.0788	*L*_11_	250.16	0.1326
*T*_12_	466.16	0.1636	*L*_12_	248.03	0.1171
*T*_13_	356.88	0.0927	*L*_13_	239.32	0.1010
*T*_14_	348.95	0.1483	*L*_14_	190.2	0.0959
*T*_15_	274.79	0.1067	*L*_15_	163.84	0.4813
*T*_16_	250.04	0.0857	*L*_16_	166.42	0.0989
*T*_17_	188.77	0.1366	*L*_17_	131.63	0.1190
*T*_18_	119.57	0.0753	*L*_18_	108.96	0.0903
*T*_19_	111.57	0.1185	*L*_19_	101.89	0.0982
*T*_20_	70.909	0.1606	*L*_20_	85.673	0.2174

The multiobjective design approach for the *H*_2_/*H*_∞_ synthetic gene network based on promoter libraries selects an adequate promoter set *c* = [*c*_1_, *c*_2_, *c*_3_] from corresponding promoter libraries such that the following two design objectives are achieved simultaneously [56]:

(i) *H*_∞_ desired noise attenuation level *ρ_d_*:

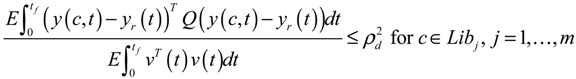
(3.23)


(ii) *H*_2_ optimal reference tracking:

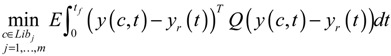
(3.24)


**Figure 12:**
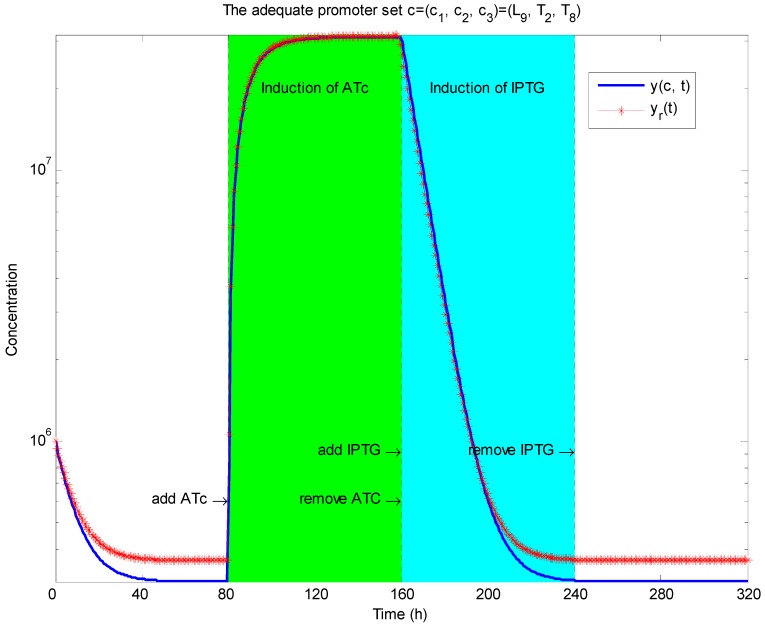
Simulation of toggle switch. By solving the LMI-constrained optimization problem of the *H*_2_/*H*_∞_ design objective Equations (3.23) and (3.24) for the synthetic gene network in Figure 11 through the library searching method, an adequate promoter set *c* = [*c*_1_, *c*_2_, *c*_3_] = [*L*_9_, *T*_2_, *L*_8_] is selected from the corresponding promoter libraries. The inducer ATc is added to the synthetic gene network at 80–160 hours to induce the gene network, and then the inducer IPTG is added at 160–240 hours. The output *y*(*c*, *t*) clearly produces a robust track with the desired reference output *y_r_*(*t*).

By solving an optimization problem with two constraints [56], adequate promoters can be selected from the corresponding libraries in Table 2 to achieve the two design objectives in Equations (3.23) and (3.24). Based on the synthetic toggle switch (Figure 11) and dynamic model (equation (3.22)) with intrinsic parameter variation and external disturbance, the adequate promoter set *c* = [*c*_1_, *c*_2_, *c*_3_] [*L*_9_, *T*_2_, *L*_8_] is selected from promoter libraries in Table 2 to achieve the multi-objective *H*_2_/*H*_∞_ reference tracking specified in Equations (3.23) and (3.24). Simulation results with *ν*(*t*) = 10 × [*n*_1_⋯*n*_6_]*^T^* are shown in Figure 12.

**Remark 4**: Collective rhythms of GRNs, especially the synchronization of dynamic cells mediated by intercellular communication, have become a subject of considerable interest to biologists and theoreticians [90]. Synchronization of a population of synthetic genetic oscillators is an important consideration in practical applications, because a population distributed over different host cells needs to exploit molecular phenomena in a simultaneous manner in order to function as a biological entity. However, this synchronization of synthetic gene networks in different host cells may be corrupted by intrinsic kinetic parameter fluctuations and extrinsic environmental molecular noise. Therefore, robust synchronization is an important design topic in nonlinear stochastic coupled synthetic genetic oscillators with intrinsic kinetic parametric fluctuations and extrinsic molecular noise. A systems biology approach indicates [59,60] that if the synchronization robustness criterion is greater than or equal to the sum of the intrinsic robustness and extrinsic robustness, then the stochastic coupled synthetic oscillators can be robustly synchronized in spite of intrinsic parameter fluctuation and extrinsic noise. If the criterion for synchronization robustness is violated, then an external control scheme can be designed to improve robustness by adding inducers to the coupled synthetic genetic network. These robust synchronization criteria and control methods are useful for a population of coupled synthetic networks with emergent synchronization behavior, especially for multicellular engineered gene networks [60].

## 4. Systems Metabolic Engineering

Systems metabolic engineering aims to amplify or delete specific genes in metabolic pathways to perform metabolic engineering within a systems biology framework. Regulatory gene networks, metabolic networks, and other cellular networks can thus be engineered in an integrated system manner. Systems biological analysis via large-scale genome-wide analyses and computational bioinformatic tools can allow the rapid evaluation of the global physiology of a cell with respect to various cellular regulations. These include transcriptional and translational regulation, as well as metabolic engineering distribution [37,61,62,63]. Results of this systems biology approach can be used to predict targets for a metabolic engineering approach within the host strain. Furthermore, the integration of high-throughput, large-scale, genome-wide analyses with in silico simulation results might provide additional information on cellular status at various hierarchical levels from genome to fluxome. Strain selection for strain improvement by systems metabolic engineering is divided into several phases [37]: (i) A base strain is allowed to develop. (ii) The base strain is further engineered via synthetic biological methods, based on results obtained from high-throughput genome-wide bioinformatic data and systems biology computational analyses. (iii) The performance of this preliminary production strain is evaluated in actual fermentation. (iv) The results are then fed back into further strain development until a superior strain showing the desired performance is derived.

By combining the results of multiple genome-wide analyses and computational analyses, systems biology may allow us to reach an unprecedented level of understanding of cellular physiology and characteristics [72], which can subsequently be used in a systems synthetic biology framework to design systems metabolic engineering strategies, for example, for strain improvement. However, novel computational methods such as large-scale data mining, multidimensional data integration, and data-driven network inference and deep curation schemes still need to be developed and applied to the integration and interpretation of heterogeneous large-scale genome-wide bioinformatic data, which are closely interconnected by complex regulatory and metabolic pathways [37,91]. Several robust biochemical circuit designs have recently been proposed to improve the network robustness of metabolic pathways. The proposed design schemes provide a systems biology method with potential applications in systems synthetic circuit design in systems metabolic engineering and systems drug design. Broadly, a metabolic network is a collection of enzymatic reactions that process cellular and intercellular metabolites. In systems metabolic engineering, the rates of reactions or fluxes, which correspond directly to changes in concentrations of substrates, enzymes, factors, or products, are often measured. Such systematic changes in concentrations can be expressed in terms of dynamic differential equations. The following S-system representation has been used for the last three decades as an efficient model for describing a dynamic metabolic network [7,27]

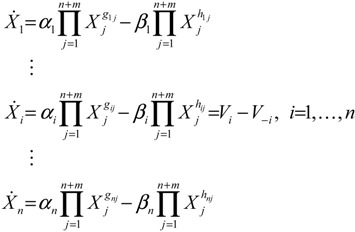
(4.1)
where *X*_1_,⋯,*X_n_*_+*m*_ are metabolites, such as substrates, enzymes, factors, or products of a biochemical network, in which *X*_1_,⋯,*X_n_* denote *n* dependent variables and *X_n+_*_1_,⋯,*X_n_*_+*m*_ denote the independent variables. In a metabolic network, intermediate metabolites and products are dependent variables, whereas substrates and enzymes are independent variables. The rate of change in *X_i_*, *Ẋ_i_* is equal to the difference between two terms, one for production or accumulation, and the other for degradation or clearance. Each term is the product of a positive rate constant *α_i_* or *β_i_* and all dependent and independent variables that directly affect production or degradation, respectively. Each variable *X_j_* is raised to the power of a kinetic parameter *g_ij_* and *h_ij_*, which represents an activating effect of *X_j_* on *X_i_* when its value is positive, and an inhibitive effect when its value is negative. *V_i_* and *V*_–_*_i_* represent aggregate flux into and out of the *X_i_* pool.

Construction of the S-system representation of a metabolic network and estimation of its kinetic parameters from experimental data are described in [7] and the literature references therein. The nonlinear parameter estimation problem of S-systems has recently been solved efficiently by evolution optimization methods [92]. It is generally difficult to study the network robustness or sensitivity of a nonlinear system such as Equation (4.1). Fortunately, many important characteristics of an S-system at or close to the steady state can be analyzed by using simple algebraic methods. Since most metabolic networks in nature operate near the steady state, at which inputs and outputs are almost balanced, the following focuses on the network robustness of metabolic networks at the steady state [7,27].

### 4.1. Robust Biochemical Circuit Design in Metabolic Networks

Consider the steady state of the metabolic network in Equation (4.1),

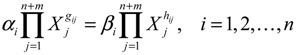
(4.2)


Taking the logarithm on both sides of Equation (4.2) and making some rearrangements,

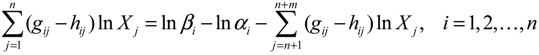
(4.3)


Introducing new variables and coefficients,

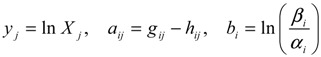
(4.4)
the steady state of the metabolic network is obtained as

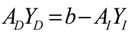
(4.5)
where

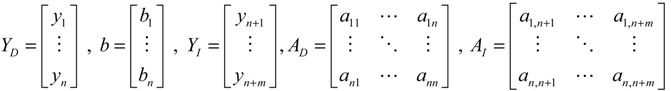



In Equation (4.5), *A_D_* denotes the system matrix of the interactions between dependent variables *Y_D_*, and *A_I_* indicates the interactions between dependent variables *Y_D_* and independent variables *Y_I_*. In the nominal parameter case, it is assumed that the inverse of *A_D_* exists so that *Y_D_* can be solved uniquely, *i.e.*, the metabolic network results in only one steady state (phenotype). Therefore, the steady state of the biochemical system is given by

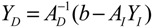
(4.6)


Suppose that parameter variations due to mutation, thermal changes, or disease can alter the kinetic properties of the steady state of a metabolic network as follows [27]

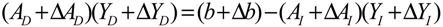
(4.7)


Parameter perturbations are defined as

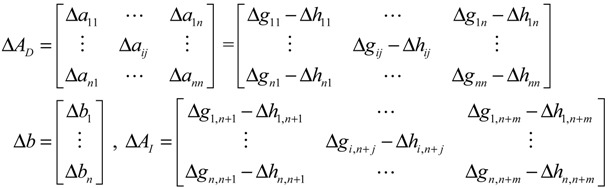

where Δ*A_D_* denotes perturbations due to kinetic parameter variations, Δ*b* denotes perturbations due to rate constant variations, and Δ*A_I_* denotes perturbations due to kinetic parameter variations between dependent and independent variables. Δ*A_D_* can influence the existence of the steady state of the metabolic network.

From Equation (4.7), we derive

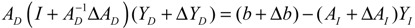
(4.8)


If the following robustness condition holds [27]

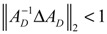
(4.9)
then the singular values of *I* + *A_D_*^−1^Δ*A_D_* are free of zero and the inverse (*I* + *A_D_*^−1^Δ*A_D_*)^−1^ exists. Therefore, the steady state of the perturbative metabolic network in Equation (4.8) is uniquely solved: as

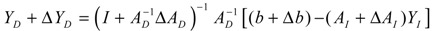
(4.10)


The above analysis implies that if the robustness condition in Equation (4.9) holds, then the steady state of a metabolic system is preserved under parameter variations Δ*A_D_*, *i.e.*, *Y_D_* + Δ*Y_D_* in Equation (4.10) has a small difference Δ*Y_D_* from the nominal in Equation (4.6) under small perturbation. However, if the condition in Equation (4.9) does not hold, then individual values of *I* + *A_D_*^−1^Δ*A_D_* may be zero, the inverse (*I* + *A_D_*^−1^Δ*A_D_*)^−1^ may not exist, and the steady state *Y_D_* + Δ*Y_D_* may cease to exist under the parameter perturbation Δ*A_D_*. As an example, consider the singular value decomposition:

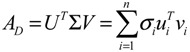
(4.11)
where *σ_i_* denotes the *i-*th singular value and *u_i_*, *v_i_*∈*R^n^* denote the corresponding left and right singular vectors, respectively. Therefore, if a parameter variation is specified as follows:

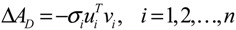
(4.12)
then

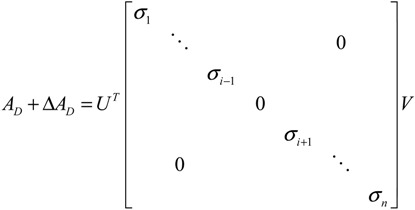
(4.13)


Obviously, the inverse (*A_D_*^−1^Δ*A_D_*)^−1^ or (*I*+*A_D_*^−1^Δ*A_D_*)^−1^ does not exist under the parameter perturbation in Equation (4.12).

**Remark 5**: The parameter perturbations in the direction of singular vectors like Equation (4.12) are the network’s points of fragility. The robustness prevents this kind of parameter variation and guarantees the existence of the steady state of the metabolic networks. When unexpected perturbations like Equation (4.12) are encountered, a catastrophic failure of the network follows. Robust circuit design is a necessary fail-safe mechanism in such situation. For example, the trehalose pathway in yeast consists of only a few metabolites that form a substrate cycle. It is governed by a surprisingly complex control system that is composed of several inhibiting or activating signaling mechanisms [7]. 

Equivalently, the network robustness of the metabolic network in Equation (4.9) can be rewritten as a more intuitive criterion for network robustness:

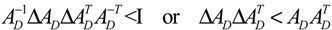
(4.14)


That is, *A_D_A_D_^T^* is the upper bound of Δ*A_D_*Δ*A_D_^T^* without violation of robust stability at steady state. If the network robustness criterion in Equation (4.14) holds, then the steady state of the perturbative metabolic network still exists.

If a metabolic network cannot tolerate perturbations, *i.e.*, the network robustness criterion in Equation (4.14) is violated, then robust control via a biochemical circuit design is a suitable remedy. Based on robustness analysis, we develop a biochemical circuit design scheme for the robust control of metabolic networks. Consider the robust control system design of the metabolic network (in Equation (4.1)) by specific biochemical feedback circuits in a more general metabolic network form [27]

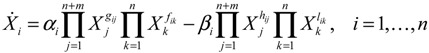
(4.15)
where 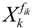 denotes a new biochemical control circuit with *X_k_* regulating the production of *X_i_* by the kinetic parameter *f_ik_*. 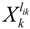
denotes a new biochemical control circuit with *X_k_* regulating the degradation of *X_i_* by the kinetic parameter *l_ik_*. The choice of regulating objects, *X_k_* and *X_i_*, and the specification of the kinetic parameters, *f_ik_* and *l_ik_*, are designed according to the feasibility of biochemical circuit linkage to achieve a desired robustness to tolerate Δ*A_D_* within the prescribed range of kinetic parameter perturbations in a metabolic network. Since *f_ik_* and *l_ik_* represent the elasticities of the corresponding enzymes in the designed control circuits, the implementation of control circuits is heavily dependent on the elasticity specification of these enzymes.

Consider the robust control system of the metabolic network in Equation (4.15). By using a similar procedure to Equations (4.2)–(4.7) at steady state,

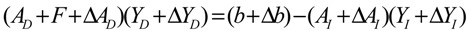
(4.16)
where *f_ij_* and *l_il_* are the kinetic parameters in *F* of the biochemical control circuit to be specified in Equation (4.15).

Suppose we can find some *F* such that the inverse of (*A_D_ + F +* Δ*A_D_*) exists. Equation (4.16) is equivalent then to

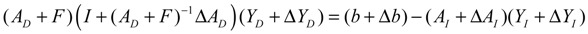
(4.17)


Similar to Equation (4.14), a robust design scheme for the controlled metabolic system in Equation (4.17) is given by

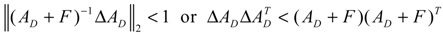
(4.18)


In this case of robust circuit design, the design purpose is to specify feedback circuits such that the structural stability robustness of the metabolic network is improved, thus enabling the network robustness criterion in Equation (4.18) to tolerate larger parameter perturbations Δ*A_D_*. The phenotype (*i.e.*, the steady state of the controlled metabolic network in Equation (4.16)) is then given by

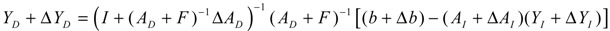
(4.19)


A simple example of network robustness analysis and circuit design is given in supplementary example 2.

### 4.2. Multipurpose Circuit Control Design of Metabolic Networks

The robustness design above focuses on the tolerance of kinetic parameter perturbations Δ*A_D_*. The effects of rate constant variations Δ*b* and of environmental changes or upstream regulatory changes Δ*Y_I_* on output variations Δ*Y_D_* should also be considered in the circuit design of metabolic networks to guarantee robustness against both intrinsic parameter variations and extrinsic environmental perturbations. The sensitivity of Δ*Y_D_* to Δ*b* in the designed metabolic network of Equation (4.16) is given by [7]

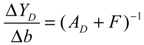
(4.20)


The sensitivity of Δ*Y_D_* to Δ*Y_I_* is given by

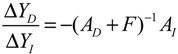
(4.21)


It is more appealing to design a robust metabolic network with desired sensitivities to variations in rate constants and environmental signals, *i.e.*,

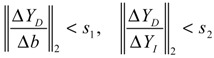
(4.22)
where the upper bounds *s*_1_ and *s*_2_ are prescribed in advance by the biochemical circuit designer. From Equations (4.20)–(4.22), the equivalent sensitivity criteria for Equation (4.22) are obtained as [27]

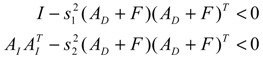
(4.23)


A multipurpose circuit design approach is therefore aimed to construct a biochemical circuit *F* to simultaneously satisfy the design requirements of network robustness in Equation (4.18) and network sensitivity in Equation (4.23). For example, suppose the goal is to design a robust biochemical circuit *f*_22_ in supplementary example 2 to tolerate kinetic parameter variation Δ*A_D_* and satisfy the network sensitivity in Equation (4.22) or (4.23) with prescribed sensitivities of ‖Δ*Y_D_*/Δ*b*‖_2_ < ‖Δ*Y_D_*/Δ*b*‖_2,nominal_ ≡ *s*_1_ = ‖*A_D_*^−1^‖_2_ = 3.42 and ‖Δ*Y_D_*/Δ*Y_I_*‖_2_ < ‖Δ*Y_D_*/Δ*Y_I_*‖_2,nominal_ ≡ *s*_2_ = ‖*A_D_*^−1^*A_I_*‖_2_ = 2.66. Then *f*_22_ should be specified to satisfy the robust circuit design and the following inequalities:

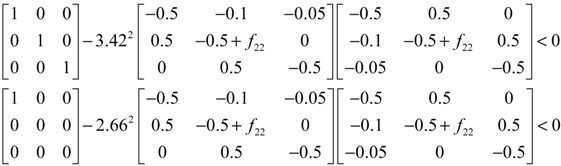
(4.24)
simultaneously. With the help of Matlab, the range of *f*_22_ necessary to tolerate Δ*A_D_* in and satisfy the desired network sensitivity in Equation (4.24) is found to be [−1, −0.081]. We choose *f*_22_ = −0.407 as a design example, which is a negative self-regulation. It has been found to efficiently eliminate the effect of parameter variations by negative compensation. About 10% of yeast genes encoding regulators are negatively self-regulating; thus, this mechanism seems to be important for maintaining robustness in yeast [27]. The metabolic network and time responses are shown in Figure S5(d) and (e), respectively.

Similarly, suppose the goal in the design case in equation is to specify *f*_12_ and *l*_22_ to satisfy equations and (4.24), *i.e.*,

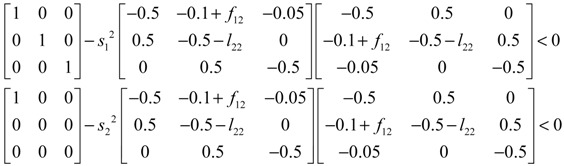
(4.25)
where *s*_1_ and *s*_2_ are the same as above. Similarly, the ranges of *f*_12_ and *l*_22_ necessary to tolerate Δ*A_D_* in equation () and satisfy the desired sensitivity criteria in Equation (4.25) are found to be [−1, 0] and [0,1], respectively. *f*_12_ = −0.08 and *l*_22_ = 0.31 are chosen as a design example. The metabolic network and time responses are shown in Figure S5(f) and (g), respectively. Another example of a TCA cycle is given in supplementary example 3.

Given that the goal is to design a robust metabolic circuit to tolerate the perturbation Δ*A_D_* and to achieve the desired sensitivities *s*_1_ and *s*_2_ in Equation (4.23), the robust control circuit design problem can be reduced to specifying *F* to satisfy the following multipurpose control circuit design derived from Equations (4.18) and (4.23):

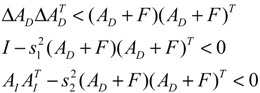
(4.26)
with nominal sensitivities *s*_1_ = ‖*A_D_*^−1^‖_2_ = 8.3685 and *s*_2_ = ‖*A_D_*^−1^*A_I_*‖_2_ = 7.5464.

If one biochemical control pathway with kinetic parameter *f*_12_ is designed to satisfy the multi-objective design criteria in Equation (4.26), then the range of *f*_12_ is found to be within [−0.8, −0.1]. If *f*_12_ is chosen as −0.2 (Figure S6(a), blue line), then the time responses of the designed TCA cycle network shown in Figure S6(d), which match the desired properties of the proposed design method, are obtained. That is, the robust controlled biochemical network not only can tolerate Δ*A_D_* (to preserve its phenotype under parameter perturbations) but also retains sensitivity to environmental molecules in the nominal case. If dynamic circuit design is employed to implement the biochemical circuit *f*_12_, then an enzyme capable of catalyzing the reaction *X*_2_→*X*_1_ is required. Additionally, a TF (*Z*) has to be found such that oxaloacetate2 (*X*_2_) can bind to the promoter of the enzyme’s inhibitor gene, as *f*_12_ is negative. The concentration of *X*_2_ could therefore regulate *X*_1_ through the kinetic parameter *f*_12_. The elasticity of the enzyme inhibitor’s gene sequence has to be modulated then to the specified performance by rational design or directed evolution. This allows the construction of the biochemical control circuit *f*_12_.

The previous section discusses robust circuit design of metabolic networks based on the steady state of an S-system model. In the following section, a stochastic dynamic model approach is taken based on the systems biology approach outlined in Section 3.

### 4.3. Robust Control Circuit Design of Stochastic Dynamic Metabolic Networks

**Figure 13:**
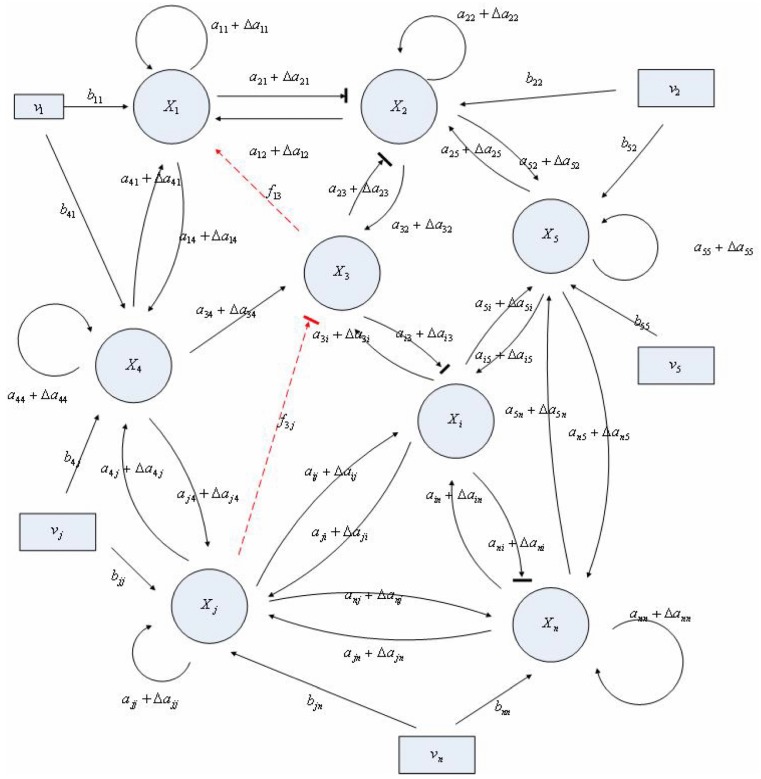
Linear metabolic network of *n* molecules with intrinsic parameter fluctuation Δ*a_ij_* and extrinsic noise *ν_i _f_ij_* denotes the biochemical circuit design from *x_j_* to *x_i_* to improve network robustness stability and noise-filtering ability.

Based on sampled data from biochemical experiments and the delayed effect of molecular diffusion and transport in cells, the linear metabolic regulatory network can be suitably modeled by the following discrete-time dynamic system:

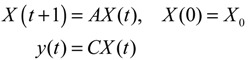
(4.27)
where the state vector *X*(*t*) denotes the discrete-time expression vector of the molecules (mRNAs, proteins, or other chemical complexes in the biochemical regulatory network) at time *t*, *A* denotes the stoichiometric (interactive) matrix among these molecules (see Figure 13), and *y*(*t*) denotes the metabolic output. Thus,

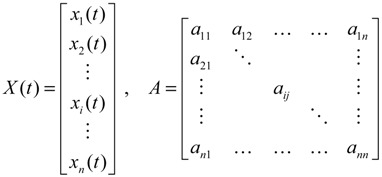

where *x_i_*(*t*), *i* = 1,⋯, *n* are molecular concentrations of mRNAs, proteins, and other complexes in the biochemical network. 

Suppose the kinetic parameters of a biochemical regulatory network of metabolic processes are affected by the following intrinsic perturbation and environmental disturbance:

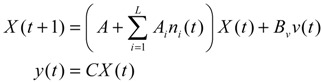
(4.28)
where 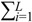 *A_i_n_i_*(*t*) denotes the intrinsic parameter fluctuations due to an *L* random fluctuation source (e.g., thermal fluctuation, alternative splicing, molecular diffusion, *etc.*), and *n_i_*(*t*) denotes the *i-*th random noise with the statistics E[*n_i_*(*t*)] = 0 and E[*n_i_*^2^(*t*)] = *σ_i_*^2^, *i* = 1,⋯, *L*. *ν*(*t*) denotes the environmental disturbance.

Based on the robust stabilization and disturbance filtering described in Section 2, the environmental disturbance attenuation level *ρ* of the metabolic network is denoted as

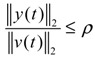
(4.29)


Following the systems biology approach in Section 2, the following robust result for a linear stochastic metabolic network is derived.

**Proposition 6** [28]:

For a linear stochastic metabolic network with intrinsic parameter fluctuation and environmental disturbance, if there exists a positive definite matrix *P* = *P^T^* > 0 such that the following matrix inequality holds for a desired disturbance attenuation level *ρ*

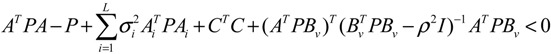
(4.30)
then the intrinsic parameter perturbation can be tolerated and environmental disturbance can be attenuated to a level *ρ* in the stochastic metabolic network Equation (4.28).

The phenotype robustness criterion in Equation (4.30) could be rewritten as

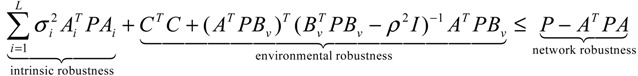
(4.31)


**Remark 6**: (i) The physical interpretation of the phenotype robustness criterion of the metabolic network in Equation (4.31) is that if intrinsic robustness allowing tolerance of intrinsic parameter fluctuation and environmental robustness, as well as attenuation of environmental disturbance are simultaneously conferred by the network robustness, then the phenotype of the metabolic network is maintained. It can be shown that if the eigenvalues of *A* are closer to the origin, then the network robustness is commensurately larger.

(ii) To solve the phenotype robustness criterion in Equation (4.30), it can be transformed into the following equivalent LMI:

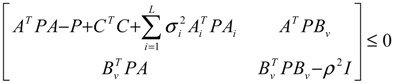
(4.32)


According to its definition, the disturbance attenuation (filtering) ability *ρ*_0_ (*i.e.*, the minimum *ρ*) can be obtained by solving the following constrained optimization problem:

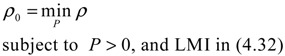
(4.33)


If the metabolic network in Equation (4.28) cannot achieve a prescribed disturbance attenuation ability *ρ_P_* (i.e., *ρ_P_* < *ρ*_0_) specified for therapeutic or biotechnological purposes, then a biochemical circuit design using state feedback is necessary:

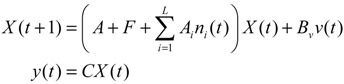
(4.34)


The phenotype robustness criterion in Equation (4.31) is then modified as

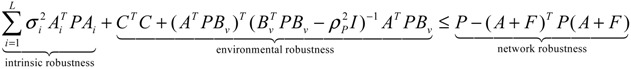
(4.35)


The negative feedback circuit *F* could shift the eigenvalues of *A* to the origin, thereby improving the network robustness of the metabolic network on the right-hand side of Equation (4.35) and achieving the prescribed attenuation level *ρ_P_* < *ρ*_0_. In general, the interactions of a biochemical regulatory network in metabolic processes are nonlinear in real biosystems. In this situation, a nonlinear biochemical regulatory network of metabolic pathways under intrinsic stochastic parameter perturbation and environmental disturbance can be represented based on the stochastic dynamic model of systems biology in Section 2:

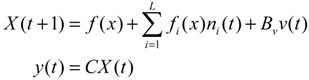
(4.36)


In the following, the robust stability and filtering ability *ρ*_0_ on *ν*(*k*) at *y*(*k*) in the stochastic nonlinear metabolic network in Equation (4.36) is discussed.

**Proposition 7** [28]:

If the following matrix inequality holds for a Lyapunov function *V*(*x*(*k*)) > 0 and a noise attenuation level *ρ*

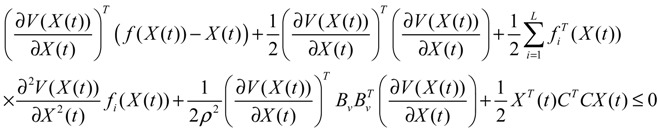
(4.37)
then the effect of environmental disturbance *ν*(*k*) on *y*(*k*) is less than *ρ*, *i.e.*, the robust disturbance attenuation level *ρ* is achieved for the nonlinear metabolic network in Equation (4.36).

The filtering ability *ρ*_0_ of the nonlinear stochastic metabolic network in Equation (4.36) that attenuates environmental disturbance can be obtained by solving the following constrained optimization problem:

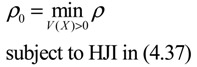
(4.38)


Following the robustness result discussed in Section 2, the phenotype robustness criterion for the nonlinear stochastic metabolic network in Equation (4.36) is given as

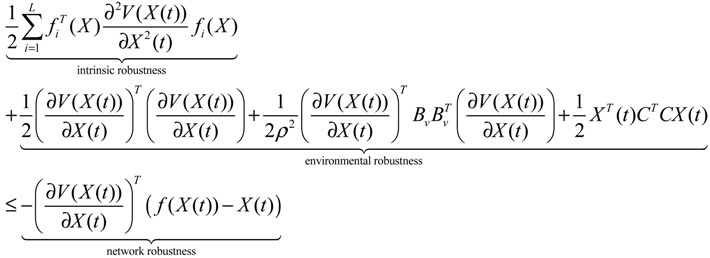
(4.39)


Suppose the network robustness of the metabolic network cannot confer enough intrinsic robustness and environmental robustness to maintain the phenotype of the metabolic network. In this situation, some negative feedback loops *Fg*(*X*(*t*)) should be implemented to improve network robustness as follows:

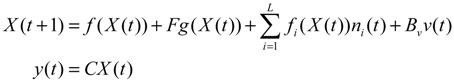
(4.40)


In this case, the phenotype robustness criterion in Equation (4.39) should be modified as follows:

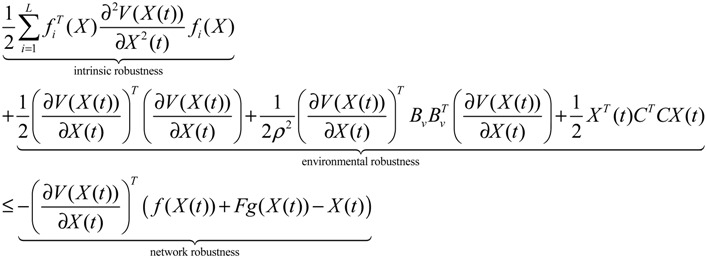
(4.41)


That is, the negative feedback loops *Fg*(*X*) are employed to improve network robustness to tolerate more fluctuations of the intrinsic parameter and to filter more environmental disturbances.

It is generally very difficult to solve the HJI in Equations (4.37), (4.39), and (4.41) for robust chemical circuit design of metabolic networks. Based on the global linearization method in Equation (3.12) [79,81] or fuzzy interpolation methods [83,93], the nonlinear stochastic metabolic network can be approximated by interpolating M local metabolic networks as follows:

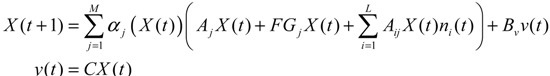
(4.42)
where *f*(*X*), *g*(*X*), and *f_i_*(*X*) are approximated by 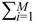 *α_j_*(*X*)*A_j_X*(*t*), 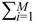 *G_j_X*(*t*), and 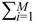 *A_ij_X*(*t*), respectively. In this situation, the following robust chemical circuit design for a metabolic network with a prescribed noise attenuation level *ρ_P_* is derived.

**Proposition 8:**

Suppose the negative feedback loop *F* is designed for the metabolic network in Equation (4.40) or (4.42), such that the following LMIs have a positive solution *P* > 0:

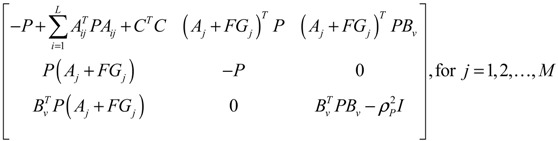
(4.43)
Thus, the prescribed disturbance attenuation level *ρ_P_* is achieved by the biochemical circuit design.

If the optimal robust filtering design is employed for the nonlinear stochastic metabolic network in Equation (4.40) or (4.42), then the control matrix *F* is specified to solve the following constrained optimization problem:

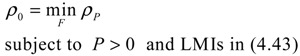
(4.44)
where *ρ_0_* in Equation (4.44) is the disturbance (noise)-filtering ability of the optimally controlled metabolic network in Equation (4.40) or (4.42) (see supplementary example 4).

This robust biochemical circuit design example in a metabolic network illustrates that the proposed systematic method of biochemical circuit design produces metabolic networks that not only tolerate more fluctuations of the intrinsic random parameter but also filter more environmental disturbances. Systems metabolic engineering design is thus capable of improving the network robustness of metabolic networks and efficiently attenuating the effect of environmental noises. This approach may serve as a basis for drug designs against genetic perturbations, pathological environmental cues (such as infectious agents or chemical carcinogens), or both.

Network robustness is a systematic property that allows a metabolic network to maintain its biochemical function or to generate biochemical products despite external disturbance and intrinsic parametric fluctuation. It is from a class of fundamental and ubiquitously observed systems-level phenomena that cannot be understood by observing individual components only. Thus, a study of network robustness at one level is simply a study of why, when, and how metabolic networks function properly or otherwise [47,80,94]. If network robustness is not large enough to obtain sufficient intrinsic robustness to tolerate random parameter fluctuations and environmental robustness to filter environmental molecular noises, then a robust biochemical circuit design is necessary to improve robustness such that the network can maintain its function or phenotype. The proposed robust circuit design principles could potentially be used for robust biosynthetic network design with applications in drug design, gene therapy, and metabolic engineering. Future focus may be on robust chemical circuit design for such applications or construction of other pathways by nanotechnology and metabolic engineering [28]. If network robustness, intrinsic robustness, and environmental robustness are taken into consideration in metabolic network engineering, such networks would function more reliably and efficiently.

**Figure 14:**
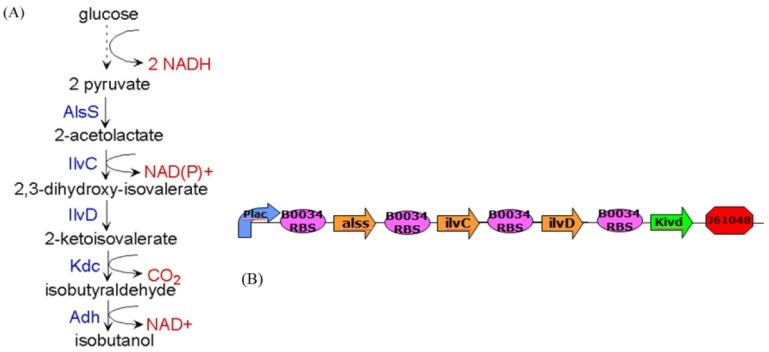
Engineered synthetic metabolic pathway for isobutanol production in *E. coli*. (**A**) Schematic representation of engineered isobutanol production pathway. (**B**) Engineered synthetic genetic circuit to generate the enzymes necessary for pathway in (**A**) for isobutanol production in *E. coli*.

With the exception of implementing feedback biochemical circuits in metabolic networks, systems synthetic biology techniques detailed in Section 3 could be combined with systems metabolic engineering approaches to engineer complete metabolic pathways or networks to produce biochemical products that could not be produced in the host cell. As an example, *E. coli* does not have non-fermentative pathways for isobutanol (see Figure 14(A)). Suppose a synthetic pathway in *E. coli* is required for the production of butanol as biofuel. In this application, it is necessary to engineer transcription and translation genes to produce the enzymes AlsS, IlvC, IlvD, and Kdc (KIVD), which are necessary to catalyze the isobutanol metabolic pathway (Figure 14(B)). The last enzyme of the metabolic pathway (Adh) is already present in *E. coli*. Promoters and RBSs with different regulatory abilities can be selected from the corresponding libraries. With adequate computation and simulation following the systems biology approach (see Section 2), a synthetic metabolic pathway could be designed and engineered (see Section 3) to achieve high-yield and high-specificity metabolic production of isobutanol.

## 5. Future Challenges in Systems Biology

Systems biology approaches to synthetic biology and metabolic engineering are built on molecular and genetic findings, and results of studies in omics fields such as genomics, proteomics, and metabolomics. The main concerns faced by systems biology are the complexity and dynamic character of biological systems, the vast quantities of biological data, and the fragmented nature of biological knowledge. These fragments of information need to be integrated with nonlinear stochastic dynamic models at different system levels by suitable computational tools before they can be applied to system synthetic biology and systems metabolic engineering.

The first challenges of systems biology are how to enrich omics data (e.g., updating and improving protein microarray, ChIP-chip databases, and pathway database for systematic studies discussed in chapters 2–4) and develop more powerful computational tools for sophisticated data handling, advanced modeling, integrated analysis, and knowledge integration. A systems biology approach based on the integration of these enriched omics data and advanced computational models will enable us to predict the behaviors of biological systems more precisely. This will increase our understanding of the underlying molecular mechanisms and our ability to efficiently predict the effects of designed genetic circuits in metabolic engineering as well as the impact of perturbations on biological systems in drug treatment.

The second challenge is how to increase the capability of researchers to navigate and relate various data and knowledge resources using the integrated platform of bioinformatics, systems synthetic biology, and system metabolic engineering to enable innovations. Connecting genomics, molecular networks, and physiology will provide us with deeper understanding of how individual differences in the genome affect physiological processes through alterations in molecular networks. Therefore, predictive and preventive medicine based on network-based biomarkers inevitably lead to personalized medicine that may revolutionize healthcare in the future [7]. The third challenge of systems biology is how to integrate bioinformatics, gene circuit design, and metabolic engineering technologies for systems drug design in future predictive and preventative medicine.

Drastically increasing oil consumption, exhaustion of natural resources, and global warming are worldwide concerns at present. They have spurred research in the areas of bioenergy and biorefining, as well as microbial production of bulk chemicals and materials. Systems biology will play an important role in these undertakings, collaborating with systems synthetic biology and systems metabolic engineering to generate bio-based products to replace some, if not most, currently used chemicals and materials. The fourth challenge of systems biology is how to make these bio-based processes competitive.

## Supplementary Materials

Supplementary materials can be accessed at: http://www.mdpi.com/2073-4409/2/4/635/s1.

Supplementary File 1 Supplementary Materials (DOCX, 1480 KB)
