# Supplementary material for: Systems Biology as an Integrated Platform for Bioinformatics, Systems Synthetic Biology, and Systems Metabolic Engineering
Source: Cells. 2013 Oct 11;2(4):635–88. doi: 10.3390/cells2040635 (PMC3972654; doi:10.3390/cells2040635)
Supplement: Supplementary File 1 — Supplementary Materials (DOCX, 1480 KB) [file cells-02-00635-s001.docx]

**Supplementary Materials**

**Figure S1. Schematic diagram of the integrated cellular network.** The construction of integrated GRN and PPI cellular networks consists of two steps. The first step is to construct the potential GRN and PPI networks by data mining. The second step is to construct real cellular GRN and PPI networks by dynamic modeling of microarray and protein expression data, respectively, via parameter identification methods (*i.e.*, reverse-engineering methods). System order detection is also employed to prune false positives in the potential cellular network to obtain a GRN and PPI integrated network. The integrated cellular network of *S. cerevisiae* under hyperosmotic stress is shown in **Figure 3**.


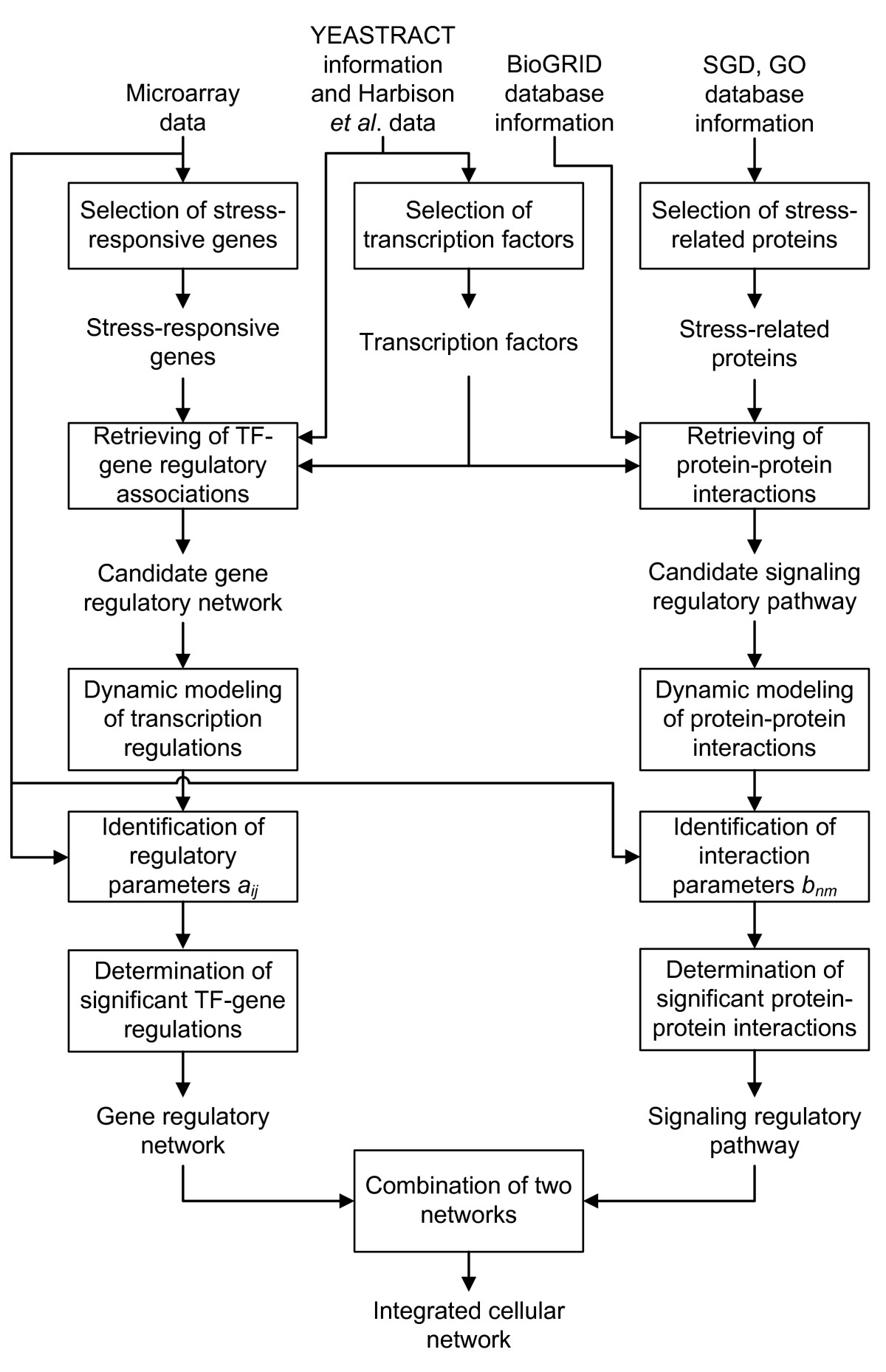


**Figure S2.** Flowchart for constructing a network-based biomarker for lung cancer investigation and diagnosis. By using microarray data for smokers with and without cancer, PPI networks with and without cancer are identified (Figure 5) through Equation (2.17). The network-based biomarker is obtained from the difference in network structure between the two networks (Figure 6). Significant proteins are determined from their carcinogenesis relevance value (CRV) through Equations (2.20) and (2.21) (Table S1).


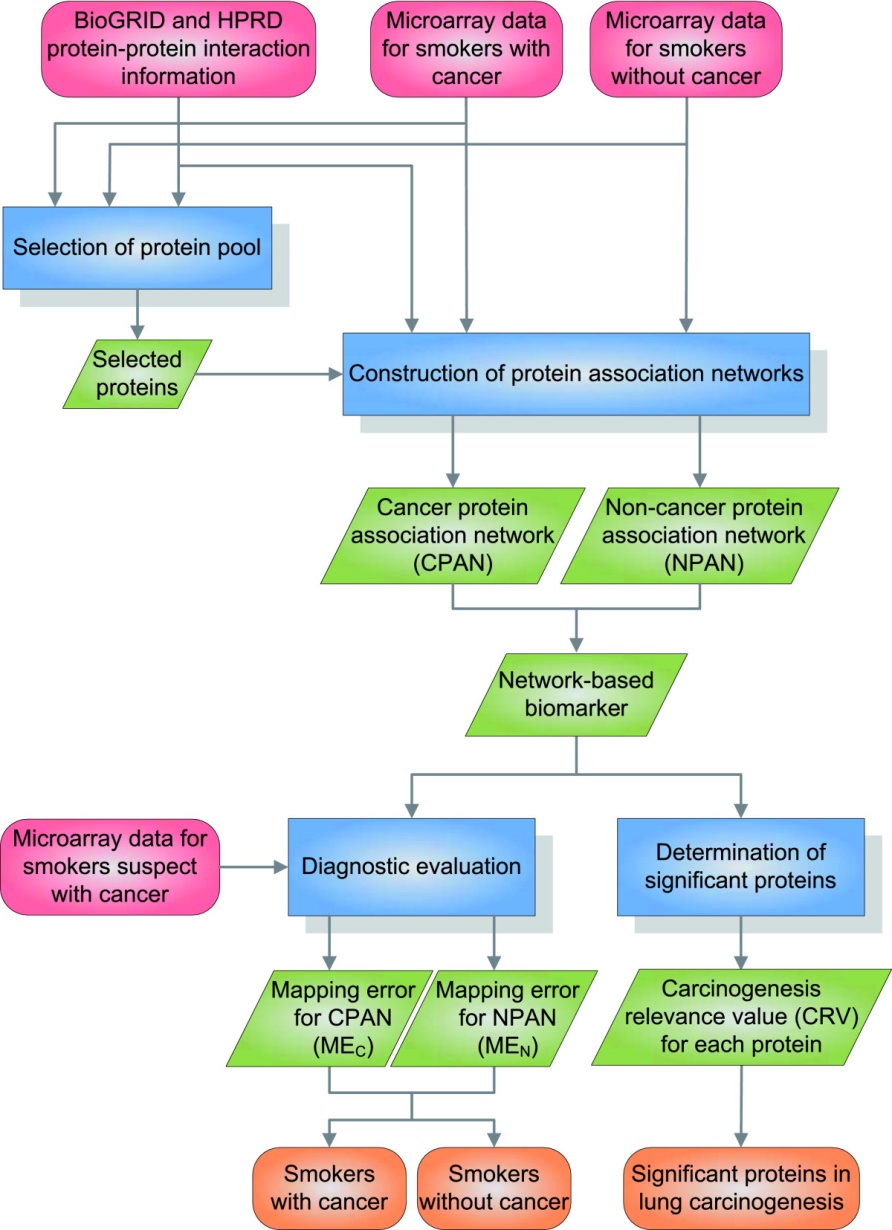


**Table S1.** Identified significant proteins in lung carcinogenesis

| **Protein symbol ^†^** | **CRV** | **P-value** | **Functional annotation *** | | |
| --- | --- | --- | --- | --- | --- |
|  |  |  | Cell growth | Cell survival | Cell migration |
| MAPK1 | 8.3418 | < 1e-5 | + | + | + |
| SMAD2 | 7.7901 | < 1e-5 | + | + | + |
| CREBBP | 5.7870 | 0.00002 | + |  |  |
| EGFR | 4.3635 | 0.00086 | + | + | + |
| AR | 4.0966 | 0.00159 | + | + | + |
| UBC | 4.0331 | 0.00180 |  |  |  |
| SRC | 3.9446 | 0.00218 | + | + | + |
| FGFR1 | 3.9227 | 0.00237 | + |  | + |
| BRCA1 | 3.9049 | 0.00243 | + | + |  |
| ESR1 | 3.8409 | 0.00295 | + | + | + |
| INSR | 3.7946 | 0.00329 | + |  | + |
| PTK2 | 3.6758 | 0.00432 | + | + | + |
| HSP90AA1 | 3.6732 | 0.00436 | + | + | + |
| CALM1 | 3.6363 | 0.00482 |  | + |  |
| POLR2A | 3.5701 | 0.00547 |  |  |  |
| CSNK2A1 | 3.4128 | 0.00761 | + | + |  |
| PRKACA | 3.3688 | 0.00856 |  | + |  |
| CTNNB1 | 3.2935 | 0.00994 | + | + | + |
| SP1 | 3.2397 | 0.01133 | + | + |  |
| SMAD4 | 3.1947 | 0.01266 | + | + | + |
| E2F1 | 3.1382 | 0.01407 | + | + |  |
| YWHAZ | 3.1212 | 0.01467 | + |  |  |
| MEPCE | 3.0968 | 0.01545 |  |  |  |
| AKT1 | 3.0193 | 0.01857 | + | + | + |
| PLCG1 | 2.9654 | 0.02069 |  |  | + |
| MYC | 2.8987 | 0.02385 | + | + |  |
| MAPK3 | 2.8545 | 0.02654 | + | + | + |
| NCOA6 | 2.8132 | 0.02892 | + | + |  |
| FYN | 2.7833 | 0.03089 | + |  | + |
| MAPK8IP3 | 2.7746 | 0.03141 |  |  | + |
| YWHAQ | 2.7582 | 0.03242 | + |  |  |
| TRAF6 | 2.7150 | 0.03535 |  | + |  |
| SMAD1 | 2.6940 | 0.03697 | + | + | + |
| SMAD3 | 2.6815 | 0.03815 | + | + | + |
| MAPK14 | 2.6727 | 0.03894 | + | + | + |
| TP53 | 2.6522 | 0.04056 | + | + | + |
| XRCC6 | 2.6270 | 0.04263 |  | + |  |
| EZR | 2.6213 | 0.04314 |  |  | + |
| TSC2 | 2.6116 | 0.04401 | + | + | + |
| HGS | 2.5730 | 0.04744 | + |  |  |

^†^ Full protein names according to UniProt database http://www.uniprot.org/webcite. * Functional annotations taken from the Gene Ontology database http://www.geneontology.org/webcite and literature.

Supplementary Example 1

An *in silico* design example for a robust synthetic gene network to confirm the robust stabilization and performance in environmental disturbance attenuation of the proposed design method for a synthetic gene network is introduced here. The synthetic gene network is shown in **Figure S3**. The goal is to synthesize a cascade loop of transcriptional inhibitions built in *E. coli* [51]. It consists of four genes, *tetR*, *lacI*, *cI*, and *eyfp*, which code for three repressor proteins, TetR, LacI, CI, and the fluorescent protein EYFP, respectively. The measured output is the fluorescence caused by the protein EYFP. The regulatory dynamic equations of the synthetic transcriptional cascade (**Figure S3**) are as follows [51].

(SE1.1)

where *κ_tetR_*_,0_, *κ_lacI_*_,0_, *κ_cI_*_,0_, and *κ_eyfp,_*_0_ are the nominal generating ratios with stochastic parameter fluctuations for the corresponding proteins, which are assumed to be 150, 587, 210, and 3487, respectively. *κ_tetR_*, *κ_lacI_*_,_, *κ_cI_*, and *κ_eyfp_* are the kinetic parameters, and *γ_tetR_*, *γ_lacI_*, *γ_cI_*, and *γ_eyfp_* are the decay rates of the corresponding proteins, which are also subject to parameter fluctuations in the host cell (*i.e.*, *E. coli*) and are specified such that they meet the four design specifications. *r_tetR_*(*x*), *r_lacI_*(*x*), *r_cI_*(*x*), and *r_eyfp_*(*x*) are the Hill functions for the repressors. They have the form *r_i_*(*x*) = *β*/[1 + (*x*/*k_i_*)*^n^*], with *β* = 1, *n* = 2, *k_i_* = 1000, and *i* = *tetR*, *lacI*, *cI*, *eyfp* [89].

The stochastic synthetic gene network of equation (SE1.1) with four random parameter fluctuation sources *in vivo* in Equation (3.7) can be represented by

$\begin{matrix} \left[\begin{matrix} dx_{tetR} \\ dx_{lacI} \\ dx_{cI} \\ dx_{eyfp} \end{matrix}\right]=\left(\left[\begin{matrix} \kappa_{tetR,0} & -\gamma_{tetR} & 0 & 0 & 0 & \kappa_{tetR} & 0 & 0 & 0 \\ \kappa_{lacI,0} & 0 & -\gamma_{lacI} & 0 & 0 & 0 & \kappa_{lacI} & 0 & 0 \\ \kappa_{cI,0} & 0 & 0 & -\gamma_{cI} & 0 & 0 & 0 & \kappa_{cI} & 0 \\ \kappa_{eyfp,0} & 0 & 0 & 0 & -\gamma_{eyfp} & 0 & 0 & 0 & \kappa_{eyfp} \end{matrix}\right]\left[\begin{matrix} 1 \\ x_{tetR} \\ x_{lacI} \\ x_{cI} \\ x_{eyfp} \\ r_{tetR}\left(x_{cI}\right) \\ r_{lacI}\left(x_{tetR}\right) \\ r_{cI}\left(x_{lacI}\right) \\ r_{eyfp}\left(x_{cI}\right) \end{matrix}\right]\right.\left.+\left[\begin{matrix} v_{1} \\ v_{2} \\ v_{3} \\ v_{4} \end{matrix}\right]\right)dt \\ +\left[\begin{matrix} \Delta\kappa_{tetR,0} & -\Delta\gamma_{tetR} & \Delta\kappa_{tetR} \\ 0 & 0 & 0 \\ 0 & 0 & 0 \\ 0 & 0 & 0 \end{matrix}\right]\left[\begin{matrix} 1 \\ x_{tetR} \\ r_{tetR}\left(x_{cI}\right) \end{matrix}\right]dw_{1}+\left[\begin{matrix} 0 & 0 & 0 \\ \Delta\kappa_{lacI,0} & -\Delta\gamma_{lacI,0} & \Delta\kappa_{lacI} \\ 0 & 0 & 0 \\ 0 & 0 & 0 \end{matrix}\right]\left[\begin{matrix} 1 \\ x_{lacI} \\ r_{lacI}\left(x_{tetR}\right) \end{matrix}\right]dw_{2} \\ +\left[\begin{matrix} 0 & 0 & 0 \\ 0 & 0 & 0 \\ \Delta\kappa_{cI,0} & -\Delta\gamma_{cI} & \Delta\kappa_{cI} \\ 0 & 0 & 0 \end{matrix}\right]\left[\begin{matrix} 1 \\ x_{cI} \\ r_{cI}\left(x_{lacI}\right) \end{matrix}\right]dw_{3}+\left[\begin{matrix} 0 & 0 & 0 \\ 0 & 0 & 0 \\ 0 & 0 & 0 \\ \Delta\kappa_{eyfp,0} & -\Delta\gamma_{eyfp} & \Delta\kappa_{eyfp} \end{matrix}\right]\left[\begin{matrix} 1 \\ x_{eyfp} \\ r_{eyfp}\left(x_{cI}\right) \end{matrix}\right]dw_{4} \end{matrix}$ (SE1.2)

Four kinetic parameters, *κ_tetR_*, *κ_lacI_*, *κ_cI_*, and *κ_eyfp_*, and four decay rates, *γ_tetR_*, *γ_lacI_*, *γ_cI_*, and *γ_eyfp_*, are designed to satisfy the following four design specifications.

(i) The biologically acceptable ranges of kinetic parameters and decay rates are given by [51]

$$\begin{matrix} \kappa_{tetR} \in\left[\text{50, 5000}\right],\text{ }\kappa_{lacI}\in\left[\text{70, 7000}\right],\text{ }\kappa_{cI} \in\left[\text{75, 8000}\right],\text{ }\kappa_{eyfp}\in\left[\text{30, 30000}\right], \\ \gamma_{tetR} \in\left[\text{0.2, 4.8}\right],\text{ }\gamma_{lacI}\in\left[\text{0.02, 0.14}\right],\text{ }\gamma_{cI}\in\left[\text{0.6, 0.8}\right],\text{ }\gamma_{eyfp}\in\left[\text{0.1, 1}\right] \end{matrix}$$

(ii) The standard deviations of parameter fluctuations to be tolerated are expressed as

$$\begin{matrix} \left[\Delta\kappa_{tetR,0}, \Delta\gamma_{tetR},\Delta\kappa_{tetR}\right]=\left[\text{30, 0.3, 50}\right],\text{ }\left[\Delta\kappa_{lacI,0}, \Delta\gamma_{lacI},\Delta\kappa_{lacI}\right]=\left[\text{50, 0.3, 200}\right], \\ \left[\Delta\kappa_{cI,0}, \Delta\gamma_{cI},\Delta\kappa_{cI}\right]=\left[\text{30, 0.3, 50}\right],\text{ }\left[\Delta\kappa_{eyfp,0}, \Delta\gamma_{eyfp},\Delta\kappa_{eyfp}\right]=\left[\text{50, 0.3, 200}\right]. \end{matrix}$$

(iii) The desired steady state *x_d_* is given by [51]

$$x_{d}=\left[\begin{matrix} x_{tetR,d} \\ x_{lacI,d} \\ x_{cI,d} \\ x_{eyfp,d} \end{matrix}\right]=\left[\begin{matrix} 1000 \\ 30000 \\ 300 \\ 30000 \end{matrix}\right]$$

(iv) The prescribed attenuation level of external disturbance is specified as *ρ* = 0.3.

By solving the LMIs in Equation (3.14) under the constraints of the design specifications, we find that if the design kinetic parameters *κ_i_* and decay rates *γ_i_* of the synthetic gene network are specified by the following ranges

$\begin{matrix} \kappa_{tetR}\in\left[\text{891, 4159}\right], \kappa_{lacI}\in\left[\text{1248, 5822}\right] \\ \kappa_{cI}\in\left[\text{1422, 6652}\right], \kappa_{eyfp}\in\left[\text{5124,24906}\right] \\ \gamma_{tetR}\in\left[\text{0.97, 4.03}\right], \gamma_{lacI}\in\left[\text{0.04, 0.08}\right] \\ \gamma_{cI}\in\left[\text{0.65, 0.75}\right], \gamma_{eyfp}\in\left[\text{0.27, 0.83}\right] \end{matrix}$ (SE1.3)

then the four design specifications (i)–(iv) are satisfied.

**Figure S3. Synthetic transcriptional cascade loop.** An *in silico* design example of a synthetic transcriptional cascade loop. TetR represses *lacI*, LacI represses *cI*, and CI represses *eyfp* and *tetR*. The fluorescent protein EYFP is the output. The regulatory dynamic equations of the synthetic transcriptional cascade are described in equation (SE1.1), and its stochastic model under random parameter fluctuations and environmental disturbance is described in equation (SE1.2).


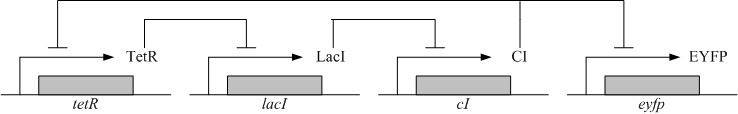


To confirm the performance of the proposed robust synthetic gene network, the network is designed by using the set of kinetic parameters *κ_i_* and decay rates *γ_i_* in the ranges in equation (SE1.3). This approach provides a test of the network’s ability to achieve the desired steady state regardless of initial conditions, parameter fluctuations, and extrinsic disturbances. The following design parameters are chosen from the ranges given in equation (SE1.3).

$\begin{matrix} \left(\kappa_{tetR},\kappa_{lacI},\kappa_{cI},\kappa_{eyfp}\right)=\left(\text{2000, 2000, 2000, 15000}\right) \\ \left(\gamma_{tetR}, \gamma_{lacI}, \gamma_{cI}, \gamma_{eyfp}\right)=\left(\text{1.98, 0.05, 0.7, 0.57}\right) \end{matrix}$ (SE1.4)

The desired steady states of the synthetic gene network *in vivo* can be achieved under intrinsic parameter fluctuations and environmental disturbances. From the in silico simulation in **Figure S4** with *v*(*t*) = [10*n_1_*, 1000*n_2_*, 10*n_3_*, 1000*n_4_*], where *n_i_*, *i* = 1,...,4 are independent Gaussian white noises with unit variance, the disturbance attenuation level of environmental disturbance, which is prescribed by *ρ* = 0.3, is estimated as

$\frac{\left(E\int_{0}^{1000} \tilde{x}^{T}Q\tilde{x}dt\right)^{1/2}}{\left(E\int_{0}^{1000} v^{T}vdt\right)^{1/2}}=0.2715<0.3$ (SE1.5)

The prescribed level of disturbance attenuation (filtering ability) is thus achieved by the proposed method. A synthetic gene network with parameters outside the ranges in equation (SE1.3) is also designed. For example, kinetic parameters *κ*_i_ and decay rates *γ_i_* may be (150, 100, 500, 1500) and (0.5, 0.05, 0.5, 0.2), respectively, which are outside the regions specified in equation (SE1.3). The simulation is shown in **Figure S4**. The time response of the synthetic network clearly suffers from more external disturbances and cannot achieve the desired steady states. In this design case, the disturbance attenuation level of external disturbance is estimated as

$\frac{\left(E\int_{0}^{1000} \tilde{x}^{T}Q\tilde{x}dt\right)^{1/2}}{\left(E\int_{0}^{1000} v^{T}vdt\right)^{1/2}}=\text{2.8093<0.3}$ (SE1.6)

The design specification for the filtering ability is significantly violated in this case.

**Figure S4.** Simulation of the example of synthetic gene network design. To confirm the stability robustness and filtering ability of the synthetic gene network in the in silico example, the synthetic gene network is simulated with initial values [200,40000,200,20000] and desired steady states [1000,30000,300,30000]. (a) With design parameters (*κ_tetR_*, *κ_lacI_*, *κ_cI_*, *κ_eyfp_*) = (2000, 2000, 2000, 15000) and (*γ_tetR_*, *γl_acI_*, *γ_cI_*, *γ_eyfp_*) = (1.98, 0.05, 0.7, 0.57) in the specified parameter range given in equation (SE1.3), the network shows sufficient stability and noise-filtering ability to achieve the desired steady state in spite of parameter fluctuations and disturbances in the host cell. (b) If the design parameters are outside the specified range, with (*κ_tetR_*, *κ_lacI_*, *κ_cI_*, *κ_eyfp_*) = (150, 100, 500, 1500) and (γ*_tetR_*, *γ_lacI_*, *γ_cI_*, *γ_eyfp_*) = (0.5, 0.05, 0.5, 0.2), then expression of the synthetic gene network shows greater fluctuation and cannot achieve the desired steady state under parameter fluctuations and environmental disturbances.


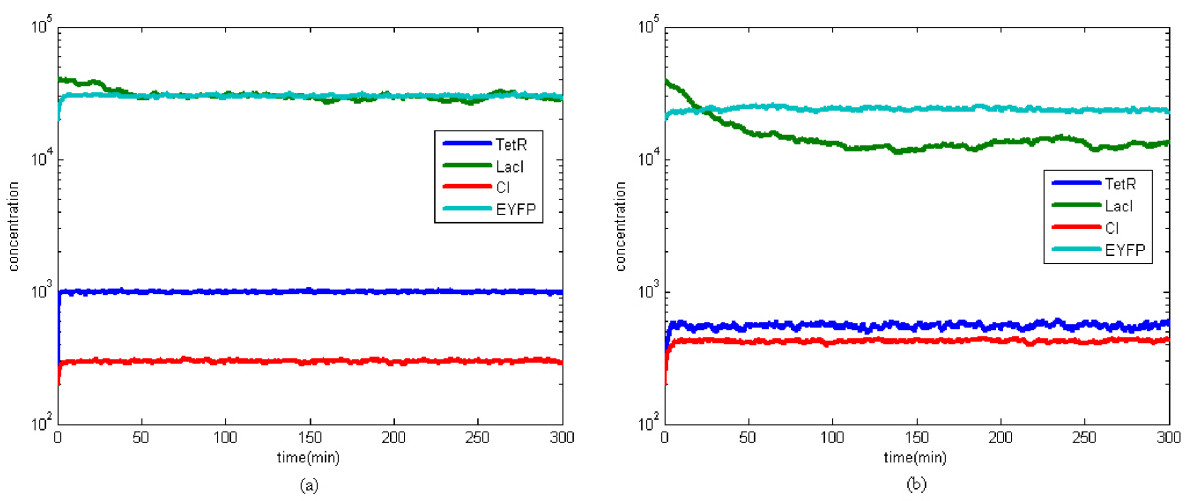


Supplementary Example 2

This provides a simple illustration of network robustness analysis and circuit design. Consider the cascaded network in **Figure S5**(a). Cascaded mechanisms are found in diverse areas of biochemistry and physiology, including hormonal control, gene regulation, immunology, blood clotting, and visual excitation [7,27]. The S-system model is given as

(SE2.1)

The time responses of the cascaded network are shown in **Figure S5**(b). Suppose the kinetic parameters *A_D_* suffer from parameter perturbations as follows:

$\Delta A_{D}=\left[\begin{matrix} 0.04675 & 0.11756 & 0.1655 \\ 0.04667 & 0.2826 & 0 \\ 0 & 0.06494 & 0.0914 \end{matrix}\right]$ (SE2.2)

System (4.19) is then perturbed as follows:

(SE2.3)

In this situation, robustness is violated and the steady state (phenotype) ceases to exist (**Figure S5**(c)). Hence, a robust circuit design is necessary to improve network robustness and tolerate this parameter perturbation. Suppose a biochemical control circuit can be designed (see **Figure S5**(d)) such that *X*_2_ can self-regulate its production to achieve the desired robustness necessary to tolerate the parameter perturbations in equation (SE2.1). The second equation in (SE2.1) can then be modified as

$X_{2}=2X_{1}^{0.5}X_{2}^{f_{22}}-1.44X_{2}^{0.5}$ (SE2.4)

**Figure S5**. Robust circuit design of the cascaded biochemical network in equation (SE2.1). (a) Cascaded biochemical network. (b) Time responses of (a) in the nominal parameter case. (c) Time responses of (a) under parameter perturbations in equation (SE2.2). (d) Designed cascaded biochemical network with *f*_22_ = −0.407 (blue dashed dotted line) following the multi-objective design in equations (SE2.5) and (4.24) in the perturbed biochemical network (SE2.3). (e) Time responses of the designed biochemical network in (d) under parameter perturbations in equation (SE2.2). (f) Designed cascaded biochemical network with *f*_12_ = −0.08 (green dashed line from *X*_2_ to the production of *X*_1_) and *l*_22_ = 0.31 (solid line ) following the multi-objective design in equations (SE2.7) and (4.25) in the cascaded metabolic network (equation (SE2.6)). (g) Time responses of the designed biochemical networks in (f) under parameter perturbations in equation (SE2.2).


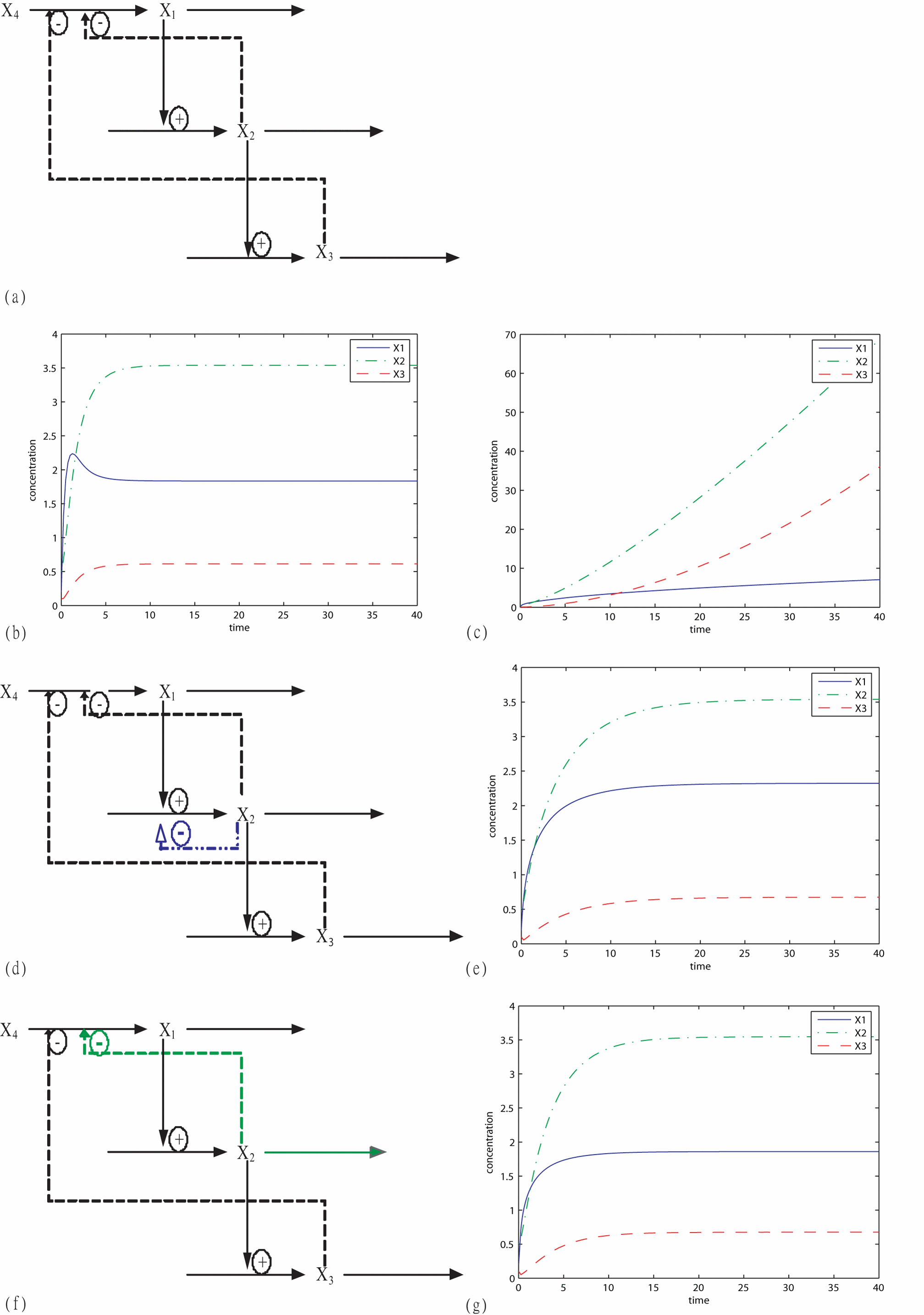


The kinetic parameter *f*_22_ should be specified in Matlab such that the robust design criterion in Equation (4.18) is satisfied. The range of *f*_22_ equired to tolerate Δ*A_D_* in equation (SE2.2) is found to be [−1, −0.081].

$\begin{matrix} \left[\begin{matrix} 0.043396 & 0.035404 & 0.022761 \\ 0.035404 & 0.082041 & 0.018352 \\ 0.022761 & 0.018352 & 0.012571 \end{matrix}\right] \\ \text{ }<\left[\begin{matrix} -0.5 & -0.1 & -0.05 \\ 0.5 & -0.5+f_{22} & 0 \\ 0 & 0.5 & -0.5 \end{matrix}\right]\left[\begin{matrix} -0.5 & 0.5 & 0 \\ -0.1 & -0.5+f_{22} & 0.5 \\ -0.05 & 0 & -0.5 \end{matrix}\right] \end{matrix}$ (SE2.5)

On the other hand, if enzyme activities can be adjusted via metabolite pathway engineering to change the kinetic parameters, an alternative design of enhancing an existing pathway by modulating its kinetic parameter value to tolerate Δ*A_D_* can be considered. For instance, suppose a catalytic control circuit can be designed such that *X*_2_ can regulate the production of *X*_1_ (*f*_12_) and *X*_2_ can self-regulate its degradation (*l*_22_; see **Figure S5**(f)) to satisfy the robust design scheme to tolerate Δ*A_D_*. The differential equations of the cascaded metabolic network in equation (SE2.1) should then be modified as follows:

(SE2.6)

The biochemical circuit design work is reduced to the manner of specifying the ranges of *f*_12_ and *l*_22_ in Equation (4.15) to simultaneously meet the robust design criterion in Equation (4.18).

$\begin{matrix} \left[\begin{matrix} 0.043396 & 0.035404 & 0.022761 \\ 0.035404 & 0.082041 & 0.018352 \\ 0.022761 & 0.018352 & 0.012571 \end{matrix}\right] \\ \text{ }<\left[\begin{matrix} -0.5 & -0.1+f_{12} & -0.05 \\ 0.5 & -0.5-l_{22} & 0 \\ 0 & 0.5 & -0.5 \end{matrix}\right]\left[\begin{matrix} -0.5 & 0.5 & 0 \\ -0.1+f_{12} & -0.5-l_{22} & 0.5 \\ -0.05 & 0 & -0.5 \end{matrix}\right] \end{matrix}$ (SE2.7)

The necessary ranges of *f*_12_ and *l*_22_ are found to be [−1, 0] and [0,1], respectively. The simulation results of the robust circuit designs with *f*_12_ = 0.08 and *l*_22_ = 0.31 for the cascaded biochemical network are shown in **Figure S5**(g).

**Supplementary Example 3**

Consider the tricarboxylic acid (TCA) cycle metabolic network in *Dictyostelium discoideum* [7]. The TCA cycle, a cyclic reaction, can produce ATP very efficiently and serve as the core of the metabolic network in most living cells. The condensation of acetyl coenzyme A (acetyl CoA) and oxaloacetic acid (OAA) results in the products citric acid and acetyl CoA. In succeeding reactions, the products cooperate with the electron-delivering mechanism and oxidative phosphorylation (ADP→ATP) at the cell membrane of prokaryotes or at the intima of eukaryotic mitochondria to oxidize an oxaloacetic acid molecule to equivalent water, CO_2_ and 12 ATP molecules. In this example, the TCA cycle mode (Figure S6(a)) is reasonably simplified to involve the following 13 dependent metabolites, 35 independent metabolites, and 26 enzyme-catalyzed processes [7,27]

| *X*_1_ | Oxaloacetate 1 (OAA 1) | *X*_25_ | Aconitase |
| --- | --- | --- | --- |
| *X*_2_ | Oxaloacetate 2 (OAA 2) | *X*_26_ | Isocitrate dehydrogenase |
| *X*_3_ | Acetyl-CoA (ACO) | *X*_27_ | Glu → Suc |
| *X*_4_ | Isocitrate (ISOC) | *X*_28_ | Aspartate transaminase |
| *X*_5_ | Pyruvate (PYR) | *X*_29_ | Alanine transaminase |
| *X*_6_ | Glutamate (GLU) | *X*_30_ | Oaa1 → Oaa 2 |
| *X*_7_ | Aspartate (ASP) | *X*_31_ | Asp → Oaa 1 |
| *X*_8_ | Alanine (ALA) | *X*_32_ | Suc → Glu |
| *X*_9_ | Citrate 1 (CIT 1) | *X*_33_ | Oaa1 → Asp |
| *X*_10_ | α-Ketoglutarate (KG1) | *X*_34_ | Protein → Asp |
| *X*_11_ | Succinate (SUC) | *X*_35_ | Protein → AcCoA |
| *X*_12_ | Fumarate (FUM) | *X*_36_ | Protein → Suc |
| *X*_13_ | Malate (MAL 1) | *X*_37_ | Protein → Fum |
| *X*_14_ | Glutamate dehydrogenase | *X*_38_ | Protein → Ala |
| *X*_15_ | α-Ketoglutarate dehydrogenase complex | *X*_39_ | Protein → Glu |
| *X*_16_ | Succinate dehydrogenase | *X*_40_ | Asp → Protein |
| *X*_17_ | Fumarase | *X*_41_ | Acetyl-CoA → Protein |
| *X*_18_ | Malate dehydrogenase | *X*_42_ | Suc → Protein |
| *X*_19_ | Malic enzyme | *X*_43_ | Fum → Protein |
| *X*_20_ | Ala → Pyr | *X*_44_ | Ala → Pro tein |
| *X*_21_ | Pyruvate dehydrogenase complex | *X*_45_ | Glu → Protein |
| *X*_22_ | Oaa 2 → Asp | *X*_46_ | NAD |
| *X*_23_ | Asp → Oaa 2 | *X*_47_ | CoA |
| *X*_24_ | Citrate synthetase | *X*_48_ | NADH |

The S-system model of the TCA cycle network in *D. discoideum* is written as follows [7]:

(SE3.1)

where

$$\begin{matrix} X_{1}\left(0\right)=0.003,X_{2}\left(0\right)=0.003,X_{3}\left(0\right)=0.065,X_{4}\left(0\right)=0.01,X_{5}\left(0\right)=0.32,X_{6}\left(0\right)=6.63 \\ X_{7}\left(0\right)=2.035,X_{8}\left(0\right)=5.313,X_{9}\left(0\right)=0.0275,X_{10}\left(0\right)=0.01,X_{11}\left(0\right)=0.9,X_{12}\left(0\right)=0.04 \\ X_{13}\left(0\right)=0.24,X_{14}=0.977,X_{15}=7610,X_{16}=3.15,X_{17}=25.7,X_{18}=77.8,X_{19}=3.08 \\ X_{20}=0.196,X_{21}=258,X_{22}=74,X_{23}=0.1,X_{24}=8.24,X_{25}=80,X_{26}=271,X_{26}=271 \\ X_{27}=0.133,X_{28}=9.95,X_{29}=2.67,X_{30}=800,X_{31}=0.1,X_{32}=1,X_{33}=74,X_{34}=1.06 \\ X_{35}=2.07,X_{36}=1.62,X_{37}=0.36,X_{38}=2.03,X_{39}=1.86,X_{40}=0.446,X_{41}=27.2,X_{42}=1.57 \\ X_{43}=7,X_{44}=0.326,X_{45}=0.24,X_{46}=0.072,X_{47}=0.1,X_{48}=0.18 \end{matrix}$$

The time responses of the TCA cycle metabolic network in equation (SE3.1) are shown in **Figure S6**(b). Suppose the metabolic network suffers an intrinsic parameter perturbation Δ*A_D_* in equation (SE3.2), which violates the upper bound of the robustness condition in Equation (4.14), so that the steady state of the TCA cycle network ceases to exist. The corresponding time responses are shown in **Figure S6**(c).

**Figure S6**. (a) TCA cycle metabolic network in *D. discoideum* redrawn from the KEGG database [7,27] The S-system model of the TCA cycle metabolic network is given in equation (SE3.1). (b) Time responses of the TCA cycle metabolic network in the nominal parameter case.


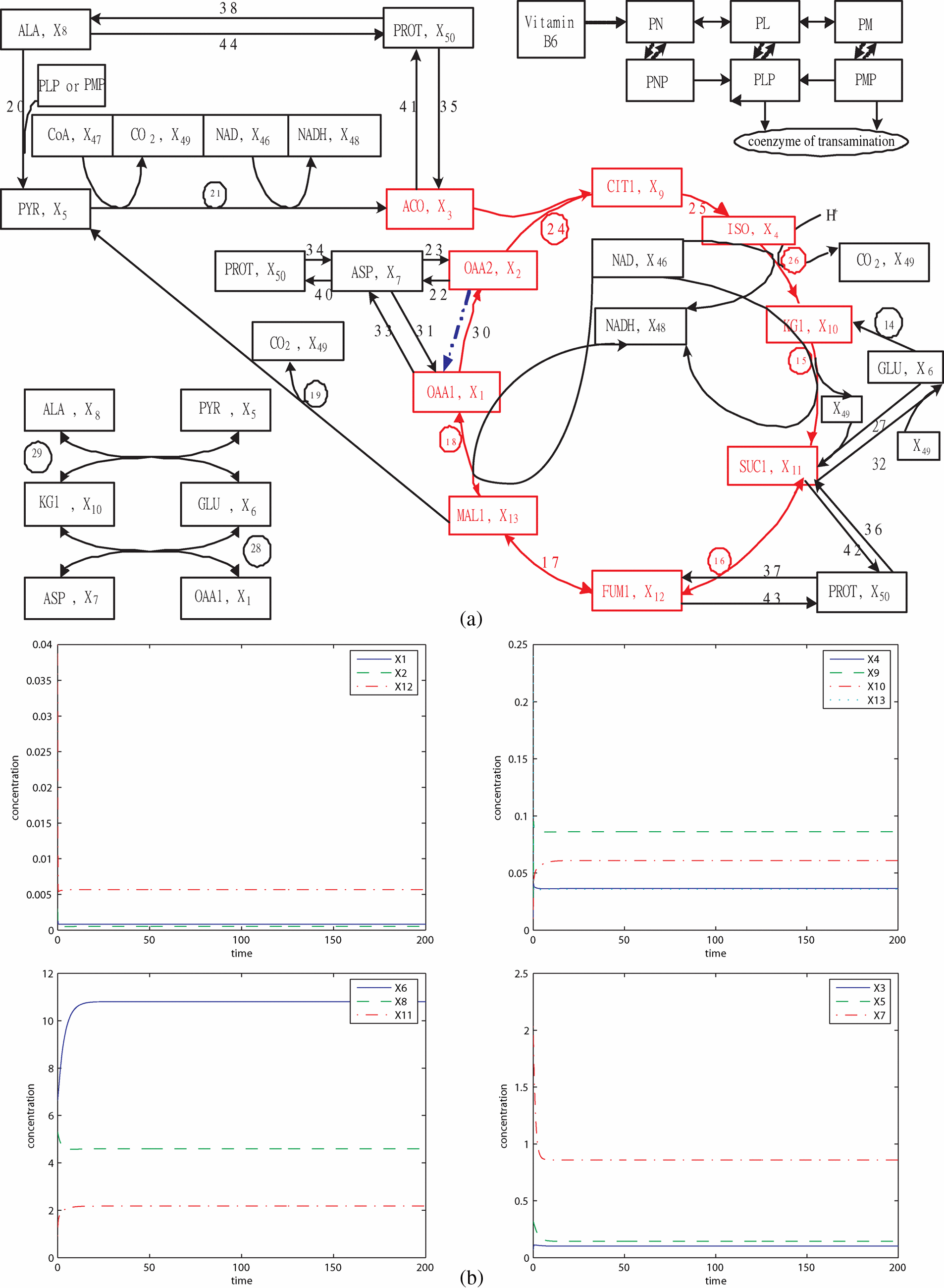


**Figure S6 – (Continued)** (c) Time responses of the TCA cycle metabolic network under parameter perturbations Δ*A_D_* in equation (SE3.2). (d) Time responses of the designed TCA cycle metabolic network with *f*_12_ = −0.2 (the dashed dotted line from *X*_2_ to *X*_1_) under parameter perturbations in equation (SE3.2).


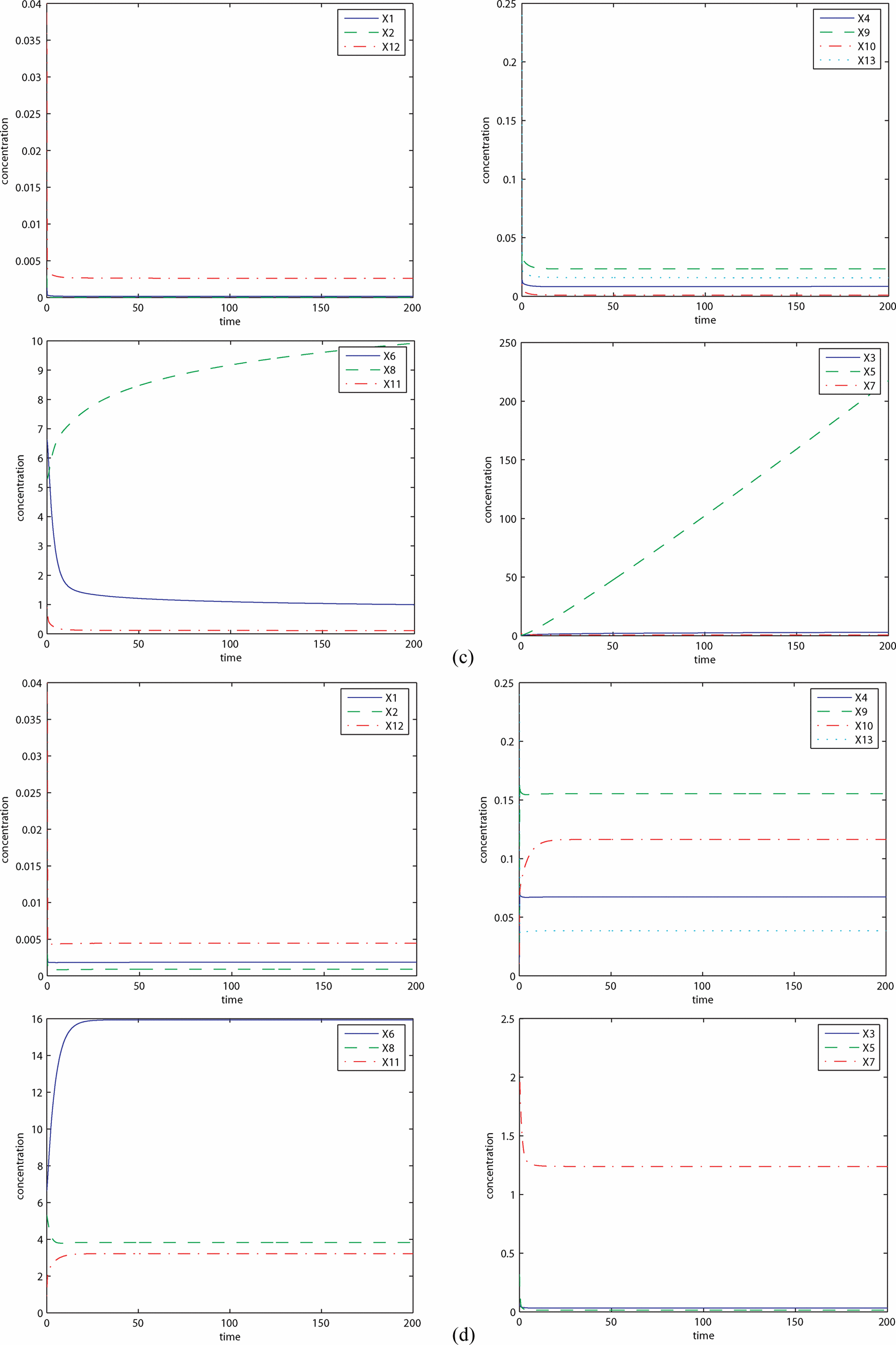


$\begin{matrix} \Delta A_{D}=\left[\begin{matrix} 0.0209 & 0 & 0 & 0 & 0 & 0.0267 & 0.000292 \\ 0.0219 & 0.0412 & 0 & 0 & 0 & 0 & 0.000305 \\ 0 & -0.0278 & 0 & 0 & 0.058 & 0 & 0 \\ 0 & 0 & 0.0157 & 0.0157 & 0 & 0 & 0 \\ 0 & 0 & 0 & 0 & 0.0611 & -0.0198 & -0.0002 \\ 0.0087 & 0 & 0 & 0 & -0.0344 & 0.0111 & 0.0001 \\ 0.00039 & 0.00726 & 0 & 0 & 0 & 0.00492 & 0.000054 \\ 0 & 0 & 0 & 0 & 0.0413 & -0.0133 & 0 \\ 0 & 0.0216 & 0 & 0 & 0 & 0 & 0 \\ 0.0119 & 0 & 0.0179 & 0.0179 & -0.0472 & 0.0153 & 0.00017 \\ 0 & 0 & 0 & 0 & 0 & 0.0207 & 0 \\ 0 & 0 & 0 & 0 & 0 & 0 & 0 \\ 0.0124 & 0 & 0 & 0 & 0 & 0 & 0.00017 \end{matrix}\right. \\ \text{ }\left.\begin{matrix} 0 & 0 & 0.0565 & 0 & 0 & 0.0142 \\ 0 & 0 & 0 & 0 & 0 & 0 \\ 0 & 0 & 0 & 0 & 0 & 0 \\ 0 & 0.0135 & 0 & 0 & 0 & 0 \\ 0.00571 & 0 & -0.0418 & 0 & 0 & -0.0105 \\ -0.0032 & 0 & 0.0235 & 0.0157 & 0 & 0 \\ 0 & 0 & 0.0104 & 0 & 0 & 0 \\ 0.0039 & 0 & -0.0282 & 0 & 0 & 0 \\ 0 & 0.0149 & 0 & 0 & 0 & 0 \\ -0.0044 & 0 & 0.0323 & 0.0215 & 0 & 0 \\ 0 & 0 & 0.0438 & 0.0293 & 0.0103 & 0 \\ 0 & 0 & 0 & 0.0239 & 0.00843 & 0.009 \\ 0 & 0 & 0 & 0 & 0.0079 & 0.0084 \end{matrix}\right] \end{matrix}$ (SE3.2)

Supplementary Example 4

This example treats a real biochemical regulatory network of operons in *E. coli*, which prefers glucose as its energy source. When glucose is in short supply, it starts metabolizing lactose (**Figure S7**). Since the metabolite pathway is very complex, some assumptions are adopted to simplify the corresponding reaction: (1) The quasi-steady-state approximation applies to the concentration of mRNA. (2) Concentrations of enzymes are equal. (3) There is a sustained lactose source outside the cell. (4) There is one delay in the conversion of lactose into *E. coli*. If the above conditions hold, then this operon model can be considered as the following discrete-time dynamic system [7] for the enzyme *e*, lactose *lac*, and allolactose *a*:

$\begin{matrix} e\left[t+1\right]= 0.15+\frac{0.1a\left[t\right]}{1+a\left[t\right]}+0.85e\left[t\right] \\ a\left[t+1\right]=\frac{0.1e\left[t\right]lac\left[t\right]}{1+lac\left[t\right]}-\frac{0.1e\left[t\right]a\left[t\right]}{1+a\left[t\right]}+a\left[t\right] \\ lac\left[t+1\right]= 0.05-\frac{0.1e\left[t\right]lac\left[t\right]}{1+lac\left[t\right]}+lac\left[t\right] \end{matrix}$ (SE4.1)

**Figure S7.** Benchmark design example of *E. coli* in a metabolic network of the operons in equation (SE4.1). The metabolic network suffers from intrinsic parameter fluctuation and environmental disturbance, as shown in equation (SE4.2).


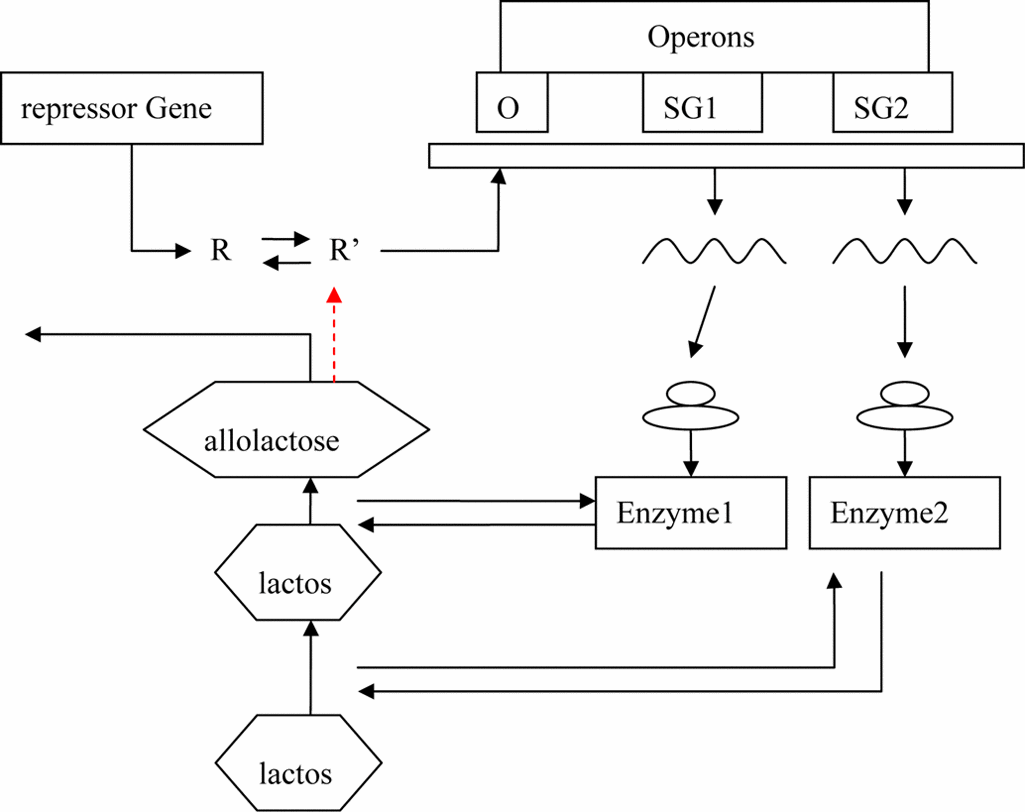


The dynamic time response in this case is shown in Figure S8(a). Suppose the operon regulatory network is also affected by intrinsic parameter fluctuations and environmental disturbance *ν*(*k*) as follows:

$\begin{matrix} e\left[t+1\right]=0.15+\frac{0.1a\left[t\right]}{1+a\left[t\right]}+0.85e\left[t\right]+n_{1}\left[t\right]\left(0.15+\frac{0.1a\left[t\right]}{1+a\left[t\right]}+0.85e\left[t\right]\right) \\ a\left[t+1\right]=\frac{0.1e\left[t\right]lac\left[t\right]}{1+lac\left[t\right]}-\frac{0.1e\left[t\right]a\left[t\right]}{1+a\left[t\right]}+a\left[t\right]+n_{2}\left[t\right]\left(\frac{0.1e\left[t\right]lac\left[t\right]}{1+lac\left[t\right]}-\frac{0.1e\left[t\right]a\left[t\right]}{1+a\left[t\right]}+a\left[t\right]\right) \\ lac\left[t+1\right]=0.05-\frac{0.1e\left[t\right]lac\left[t\right]}{1+lac\left[t\right]}+lac\left[t\right]+n_{2}\left[t\right]\left(0.05-\frac{0.1e\left[t\right]lac\left[t\right]}{1+lac\left[t\right]}+lac\left[t\right]\right)+v\left[t\right] \end{matrix}$ (SE4.2)

The stochastic intrinsic parameter fluctuations are *n*_1_[*t*], which includes transcriptional and translational noise and *n*_2_[*t*], which includes transport noise. Both are zero mean white noises with variances of *σ*_1_ = 0.1 and *σ*_2_ = 0.04. External disturbance is *ν*[*t*] = 5e^−^°^.^°^5^*^t^*(cos(0.2*πt*) + 1).

Again, the goal is to design a pathway from the final product allolactose to the regulatory gene. This design produces the corresponding enzyme to robustly stabilize allolactose production *a*(*t*). In this case of robust feedback circuit design, the design objective is based on the transfection technique. This method involves modifying the binding site of the promoter region of the corresponding gene of the control enzyme to change transcriptional ability and basal production rate. The engineered single control pathway circuit has two kinetic parameters *F*_1_ and *F*_2_ to robustly stabilize the biochemical network. The following dynamic equation has to be modified with the control terms:

$e\left[t+1\right]=0.15F_{1}+\frac{0.1a^{F_{2}}\left[t\right]}{1+a^{F_{2}}\left[t\right]}+0.85e\left[t\right]+n_{1}\left[t\right]\left(0.15+\frac{0.1a\left[t\right]}{1+a\left[t\right]}+0.85e\left[t\right]\right)$ (SE4.3)

The design parameters *F*_1_ and *F*_2_ are Michaelis constants. Since the designed steady states are kept close to the nominal ones, the relationship between *F*_1_ with *F*_2_ is *F*_1_ = 1.93014 − (0.6546*^F^*^2−1^/(1 + 0.6546*^F^*^2^)) (equation (SE4.3)). Because Michaelis constants are positive, we choose *F*_2_ > 0. Based on the global linearization scheme and the proposed robust filter design with a desired disturbance attenuation level *ρ_P_* = 1 in Equation (4.43), we choose four convex hull vertices in the form of four globally linearized systems, and set *α_j_*(*X*) = 0.25. The independent control parameter *F*_2_ is then chosen such that the following designed metabolic network with desired disturbance attenuation level *ρ_P_* = 1 is guaranteed:

$\begin{matrix} X[t]= \left[e[t] a[t] lac[t]\right]^{T} \\ f(X,F)=\left[\begin{matrix} 0.15F_{1}+\frac{0.1a^{F_{2}}\left[t\right]}{1+a^{F_{2}}\left[t\right]}+0.85e\left[t\right] \\ \frac{0.1e\left[t\right]lac\left[t\right]}{1+lac\left[t\right]}-\frac{0.1e\left[t\right]a\left[t\right]}{1+a\left[t\right]}+a\left[t\right] \\ 0.05-\frac{0.1e\left[t\right]lac\left[t\right]}{1+lac\left[t\right]}+lac\left[t\right] \end{matrix}\right] \\ f_{1}(X)=\left[\begin{matrix} 0.15+\frac{0.1a\left[t\right]}{1+a\left[t\right]}+0.85\left[t\right] & 0 & 0 \end{matrix}\right]^{T} \\ f_{2}(X)=\left[0 \frac{0.1e[t]lac[t]}{1+lac[t]}+a[t]-\frac{0.1e[t]a[t]}{1+a[t]}\right.\text{ }\left.0.05+lac[t]-\frac{0.1e[t]lac[t]}{1+lac[t]}\right]^{T} \\ B_{v}=[0 0 0.1]^{T}, C_{Z}=[0 1 0]\text{ } \end{matrix}$ (SE4.4)

With the help of the LMI toolbox, we found that *F*_2_ ∈ (1,∞) could satisfy the LMIs in Equation (4.43) to guarantee *ρ_P_* = 1. For convenience of design, let *F*_2_ = 2. The corresponding control kinetic parameters are [*F*_1_ *F*_2_] = [2.0968 2]. The solution *P* for the robust biochemical circuit design in Equation (4.43) for the prescribed disturbance attenuation level *ρ_P_* = 1 is given by

$P=\left[\begin{matrix} 0.5397 & -1.1067 & 0 \\ -1.1067 & 1504.3942 & 0 \\ 0 & 0 & 0.01 \end{matrix}\right]>0$ (SE4.5)

**Figure S8.** (a) The dynamic time response of each molecule of the nominal biochemical regulatory system in equation (SE4.1). (b) Comparison of the time response of allolactose (*a*(t)) of the designed metabolic system with the time responses of the nominal metabolic system and perturbed metabolic system. The proposed robust circuit design tolerates intrinsic fluctuation and significantly improves the filtering of extrinsic noise.

**
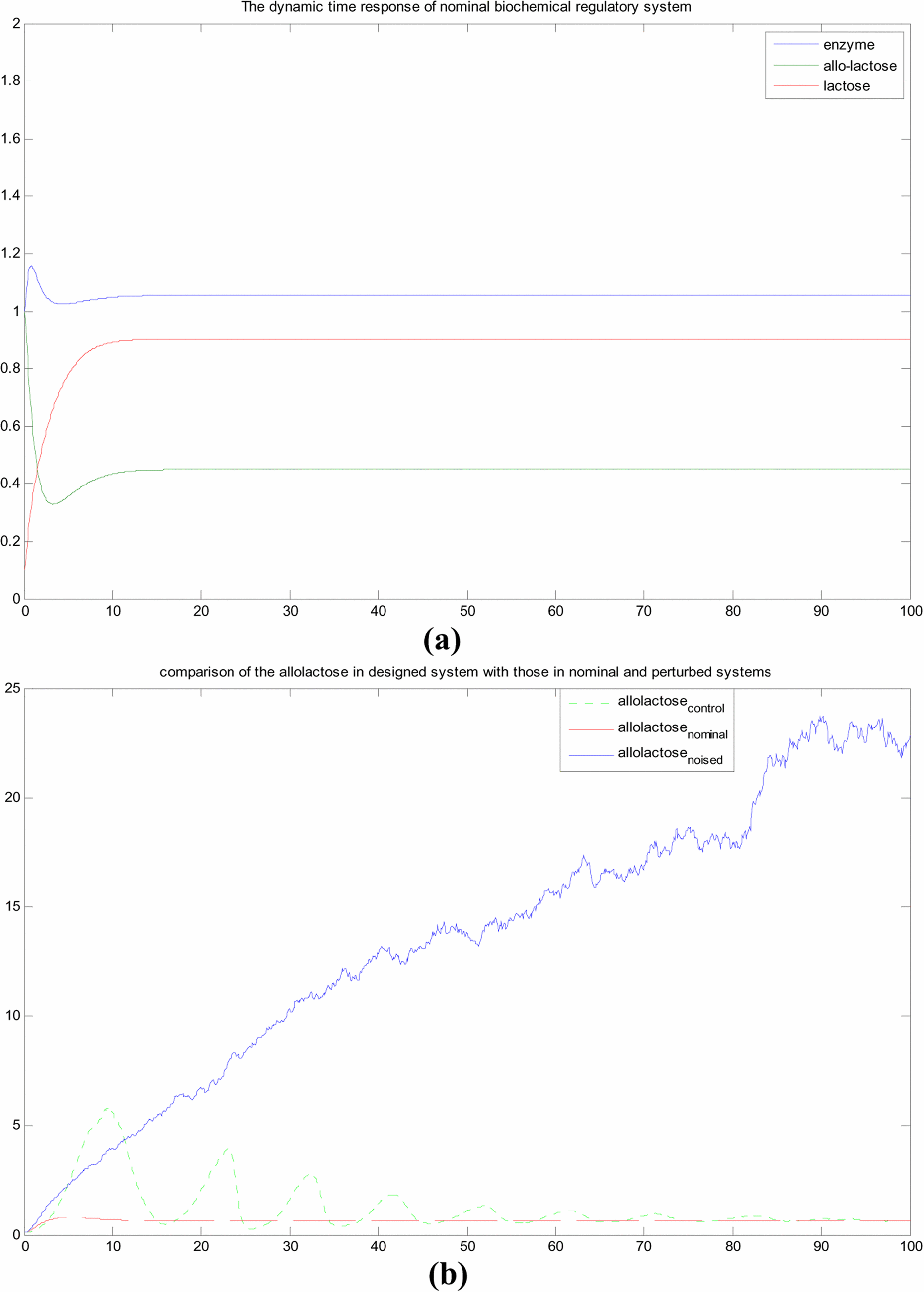
**

Figure S8(b) shows the simulation results. The network filtering ability can be calculated as *ρ* ≈ 0.6814 < 1 *= ρ_P_*. The result of the theoretical attenuation level is clearly more conservative. However, using the proposed robust method of biochemical circuit design, the prescribed disturbance attenuation level *ρ_P_* ≤ 1 can be guaranteed for the metabolic network. The conservative result is mainly due to the conservativeness of both Lyapunov stability and LMIs in the design procedure.
